# Comparison of the broncoalveolar lavage fluid proteomics between foals and adult horses

**DOI:** 10.1371/journal.pone.0290778

**Published:** 2023-09-05

**Authors:** Alejandra A. Rivolta, Adina R. Bujold, Phillip A. Wilmarth, Brett S. Phinney, Joseph P. Navelski, David W. Horohov, Macarena G. Sanz

**Affiliations:** 1 Department of Veterinary Clinical Sciences, College of Veterinary Medicine, Washington State University, Pullman, Washington, United States of America; 2 Department of Pathobiology, University of Guelph, Guelph, Ontario, Canada; 3 Proteomic Shared Resource, Oregon Health & Science University, Portland, Oregon, United States of America; 4 Genome Center Proteomics Core Facility, UC Davis, Davis, California, United States of America; 5 School of Economic Sciences, Washington State University, Pullman, Washington, United States of America; 6 Gluck Equine Research Center, University of Kentucky, Lexington, Kentucky, United States of America; University College London, UNITED KINGDOM

## Abstract

Neonates have different cellular composition in their bronchoalveolar lavage fluid (BALF) when compared to foals and adult horses; however, little is known about the non-cellular components of BALF. The objective of this study was to determine the proteomic composition of BALF in neonatal horses and to compare it to that of foals and adult horses. Bronchoalveolar lavage fluid samples of seven neonates (< 1 week age), four 5 to 7-week-old foals, and six adult horses were collected. Quantitative proteomics of the fluid was performed using tandem mass tag labeling followed by high resolution liquid chromatography tandem mass spectrometry and protein relative abundances were compared between groups using exact text. A total of 704 proteins were identified with gene ontology terms and were classified. Of these, 332 proteins were related to the immune system in neonates, foals, and adult horses. The most frequent molecular functions identified were binding and catalytic activity and the most common biological processes were cellular process, metabolic process, and biological regulation. There was a significant difference in the proteome of neonates when compared to foals and to adult horses. Neonates had less relative expression (FDR < 0.01) of many immune-related proteins, including immunoglobulins, proteins involved in the complement cascade, ferritin, BPI fold-containing family B member 1, and macrophage receptor MARCO. This is the first report of equine neonate BALF proteomics and reveals differential abundance of proteins when compared to BALF from adult horses. The lower relative abundance of immune-related proteins in neonates could contribute to their susceptibility to pulmonary infections.

## Introduction

The pulmonary immune system of equine neonates is not mature at birth [1]. This may explain why they are susceptible to pathogens such as *Rhodococcus equi* (*R*. *equi*) infection while older foals are less susceptible and adult horses are resistant to infection with this bacterium unless they are immunocompromised [2–4]. The underlying mechanism for age resistance to this and other pathogens remains poorly understood and evaluation of the neonatal respiratory immune system has been mostly limited to the cellular component of bronchoalveolar lavage fluid (BALF) [2, 5, 6]. Equine neonate BALF is comprised of ~ 80% macrophages, 5–20% lymphocytes and ~ 9% of neutrophils. This differs from equine adult BALF that has 40–65% of macrophages, 30–70% of lymphocytes and 5% of neutrophils [7, 8]. The bactericidal activity of macrophages [2] and the phagocytic capacity of neutrophils [9] is lower in foals than in adult horses. As foals age, the percentage of macrophages decreases and the percentage of lymphocytes increases until they reach adult levels by 3 months of age [8]. Neutrophil activity and numbers reach adult levels at 3–4 weeks of age [9, 10].

Little is known about the non-cellular immune components of foal BALF. This fraction includes proteins, immunoglobulins, and other macromolecules such as surfactant complex. Interferon gamma (IFN-γ) production is deficient in BALF-originated lymphocytes of neonates and only reaches adult levels in yearlings [11]. Neonates are also born hypogammaglobulinemic [12] and require colostrum ingestion for passive transfer of immunoglobulins until endogenous production begins at around 1–3 months of life [13]. After the ingestion of colostrum, maternal IgG is detected in high concentrations in nasal secretions during the first 2 weeks of life but endogenous production is not seen for the first 42 days [14]. Pulmonary surfactant proteins play a role in lung protection as they modulate the inflammatory response against pathogens [15]. Surfactant analysis showed a significant difference in the composition of the phospholipids present in surfactant between neonates and adult horses [16].

While other directed protein identification techniques are limited to a small number of proteins, quantitative proteomics methods allow characterization of the whole proteome of a sample and enables for comparison of relative abundances between samples. The proteome of BALF from healthy adult horses [17] and horses with asthma [18] has been reported. To the authors’ knowledge, the proteomic analysis of the equine neonate BALF has not been reported. Age-related differences in protein composition of BALF may be important in the future understanding of the pulmonary immune system and neonatal susceptibility to pulmonary infection. The objective of this study was to determine the proteomic composition of BALF in neonate horses and to compare it to that of older foals and adult horses.

## Material and methods

### Animals and sample collection

All samples used in the present study were collected as part of a previously reported study [3] that was approved by the Institutional Animal Care and Use Committee at the University of Kentucky (ASAF 2010–0624). Seven **neonates** (<1 week age, 3 males and 4 females), four 5 to 7-week-old **foals** (sex not recorded), and six **adult** Thoroughbred horses (> 10 years old, all females) that belonged to the University of Kentucky research herd were used. The age of the foals was selected based on the fact that this age group shows little to no susceptibility to *R*. *equi* infection [3, 4]. All included animals were deemed healthy based on physical examination. All neonates and foals had adequate passive transfer (IgG > 800 mg/dL) and unremarkable thoracic ultrasonography [19]. Bronchoalveolar lavage fluid samples were collected as previously described [20]. Briefly, horses were sedated using xylazine (0.15 mg/kg IV; Butler Co., Dublin, OH) and butorphanol tartrate (0.01 mg/kg IV; Fort Dodge Animal Health, IA, USA). Thereafter, a sterile BAL tube lubricated at the distal end (8mm OD, 160cm for neonates and foals, and 10 mm OD, 300 cm for adult horses) was passed through the nasal passage, through the trachea, and into the distal airway until gently seated in a bronchiole. Luer-lock 60 mL syringes (Tyco Health Care, MA, USA) filled with sterile phosphate-buffered saline (PBS, Corning, NY, USA) were attached to the catheter on the proximal end of the inner BAL tube. The total amount of PBS infused was 100 mL for all neonates and foals and 240 mL for adult horses. The PBS was instilled into the lung and immediately withdrawn using syringe suction. Sampling of the lower airway was visually confirmed by the presence of surfactant (foam) in the fluid recovered.

### Sample preparation

Samples were centrifuged at 500 x g for 15 minutes, filtered using 0.2 μm pore filter to remove additional cellular components, and stored at -80 °C until preparation. Thereafter, they were lyophilized to further concentrate them to 75 μg/mL (largest concentration obtained from the more diluted sample). Total protein concentration was determined by BCA assay. Protein preparation was accomplished with S-trap columns (Protifi, NY, USA) according to manufacturer’s instruction and digestion into peptides with trypsin (Promega, WI, USA). Samples were stored at -80 °C until processing.

### TMT labeling

All sample peptides were labeled as previously described [21] using TMT Mass Tagging kits (Thermo Scientific, MA, USA). Briefly, peptides were resuspended in 100 μL of triethylammonium bicarbonate (TEAB). A pooled sample for normalization between runs was prepared by combining 6.25 μg of peptide from each sample. TMT labeling was accomplished on 25 μg of peptides by adding 40 μL of TMT label previously resuspended in acetonitrile. Samples were randomly distributed between 10-plex label sets with two pooled samples per set and submitted to UC Davis Proteomics Core Facility for LC-MS/MS analysis.

### Nano LC-MS/MS

The proteomics analysis was conducted at UC Davis Proteomics Core Facility (CA, USA). High resolution liquid chromatography (LC) separation was done on a Dionex nano Ultimate 3000 UPCL (Thermo Scientific, MA, USA) followed by electrospray ionization with an Easyspray source and mass spectrometry on Thermo Scientific Fusion Lumus (Thermo Scientific, MA, USA), as previously described [22]. Briefly, multiplexed TMT-labeled samples were resuspended in 5% formic acid and separated by LC. Eluted peptides were dissociated using CID fragmentation energy of 35%, turbo scan speed, 50 ms max inject time, 1x10^4^ automatic gain control (AGC) and maximum parallelizable time turned on, and fragment ions were measured in the linear ion trap. Reporter ion detection was performed in the Orbitrap mass analyzer using MS3 scans with a resolution of 50K and a scan range of 100–500 Da, 105 ms max inject time and 1x10^5^ AGC, following synchronous precursor isolation of the top 10 ions in the linear ion trap, and higher-energy collisional dissociation in the ion-routing multipole.

### Database searching

Raw data were converted to text files and compressed using MSConvert (Proteowizard 2.1, http://proteowizard.sourceforge.net). MS2 format files were created for each RAW file and reporter ion peak heights extracted using Python scripts. A canonical FASTA file (taxon = 9796, horse, version 2019.05, 21455 sequences) was downloaded from Uniprot (http://uniprot.org) in May 2019. Common contaminant sequences and sequence-reversed decoys were added and database searching was conducted using Comet (version 2016 release 3) [23]. The searches used semi-tryptic cleavage, 1.25 Da precursor mass tolerance, 1.0005 Da fragment ion tolerances, variable oxidation of methionine, and static modifications for alkylated cysteines and TMT tags (at peptide N-term and lysine). The proteomic analysis workflow (PAW) pipeline was used for peptide spectrum match (PSM) validation using the target/decoy method as previously described [24]. Basic parsimony and extended parsimony analyses were performed to obtain the final protein groups. Identified proteins were mapped to human orthologs using scripts available at https://github.com/pwilmart/PAW_BLAST. Accession numbers were transformed to Gene Ontology (GO) terms using gene ontology enrichment analysis and visualization tool (GOrilla) (http://cbl-gorilla.cs.technion.ac.il/) and classified using PANTHER (http://pantherdb.org/) software and GOrilla to facilitate data visualization.

### Statistical analysis

Internal reference scaling (IRS) used the pooled reference samples to put each TMT plex on a common reporter ion intensity scale so that all biological samples could be directly compared [21]. The pool reference sample was prepared using a pool of all the samples submitted for the present study. All statistical analyses were performed using the Bioconductor R statistical software (https://www.r-project.org/) package edgeR [25]. To compare differences in protein expression among the different groups exact test was performed [26, 27]. Benjamini-Hochberg multiple testing correction (a false discovery rate, FDR) of edgeR p-values was set at 0.01. Maximum peptide intensities were log 2 transformed to provide the protein expression levels (PELs) [28].

## Results

### IRS normalization process

The graphs before and after IRS normalization were plotted (S1 Fig) for the eight identical pooled reference samples, which demonstrated that the IRS method successfully removed the large variation from data acquisition random MS2 scan selection. The trended variance for moderated testing was calculated and a trimmed mean of M-values (TMM) normalization method was performed on the 17 samples used for this study (S2 Fig) [29].

### Proteins in BALF

**Fig 1:**
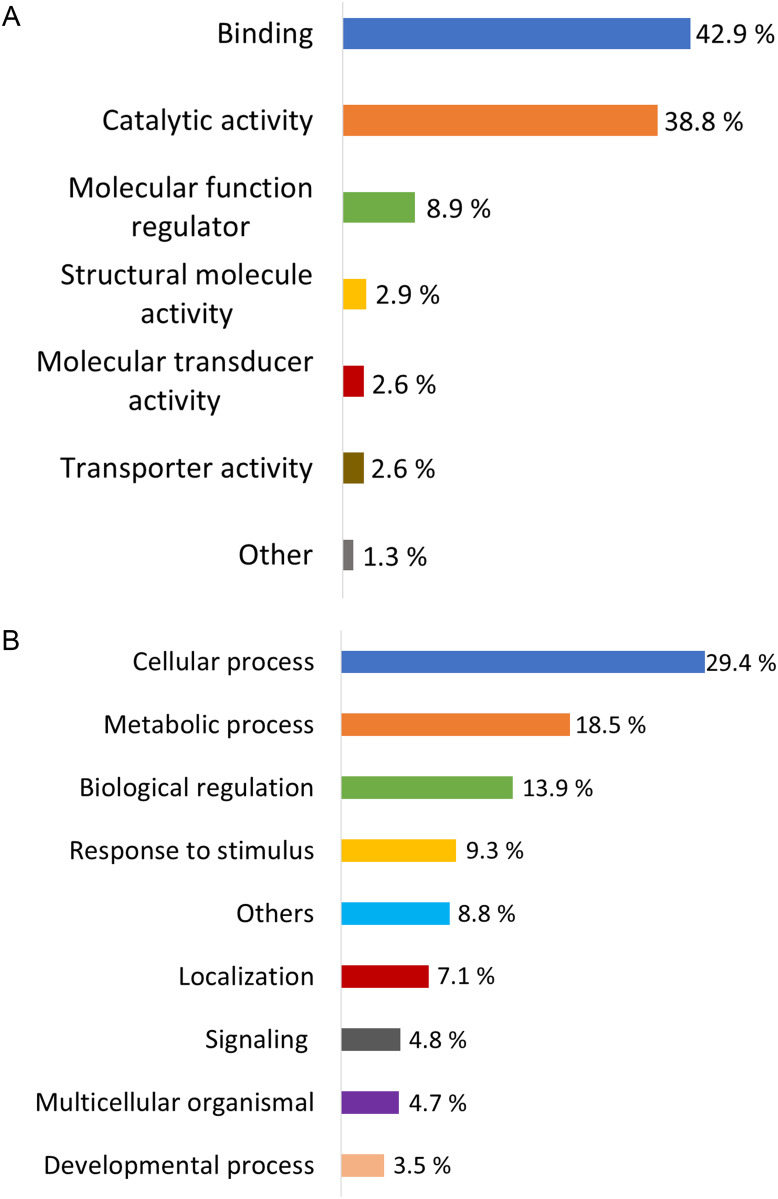
Percentage of frequency of (A) Molecular functions and (B) Biological processes of the proteins with GO terms present in all the BALF samples from neonates, foals, and adult horses. Molecular functions with < 1% of frequency and biological processes with < 3.5% of frequency were grouped under “Others” category in each histogram.

Bronchoalveolar lavage fluid from all samples were analyzed using nano LC-MS/MS. Data were combined and a total of 1,341 proteins were confidently identified. The IRS method requires protein intensity measurements for the pooled reference channels from each of the plexes and resulted in 925 proteins that could be quantified. Eight hundred and eighty-four of the quantifiable proteins had reporter ion signals present in the 17 samples and successfully converted from human ortholog Uniprot accession numbers to NCBI gi accession numbers using Uniprot retrieve tool (S1A Table). A total of 704 proteins had a GO term and were classified through their molecular function and biological process. The most predominant molecular functions present were binding (42.9%, n = 302) and catalytic (38.8%, n = 273) activities. The most common biological processes were cellular process (29.4%, n = 443) followed by metabolic process (18.5%, n = 278); 249 proteins were represented in both processes. The percentage distribution of each category was the same for all groups (Fig 1).

The number of proteins related to immune processes, according to Uniprot, were reported as a percentage of the total proteins identified. Three hundred and thirty-two proteins (37.5%) were related to immune responses in neonates, foals, and adult horses. The complete list of immune-related proteins is detailed in S2 Table.

### Comparison between neonates, foals, and adult horses

Following identification of the peptides and normalization of the results, the three groups were compared using the exact test to evaluate differences in the relative abundance level of the BALF proteins. The detailed differences in protein expression levels between groups can be seen in the heatmaps of S3 Table and are listed in S1B and S1C Table.

#### Immune-related proteins

**Fig 2:**
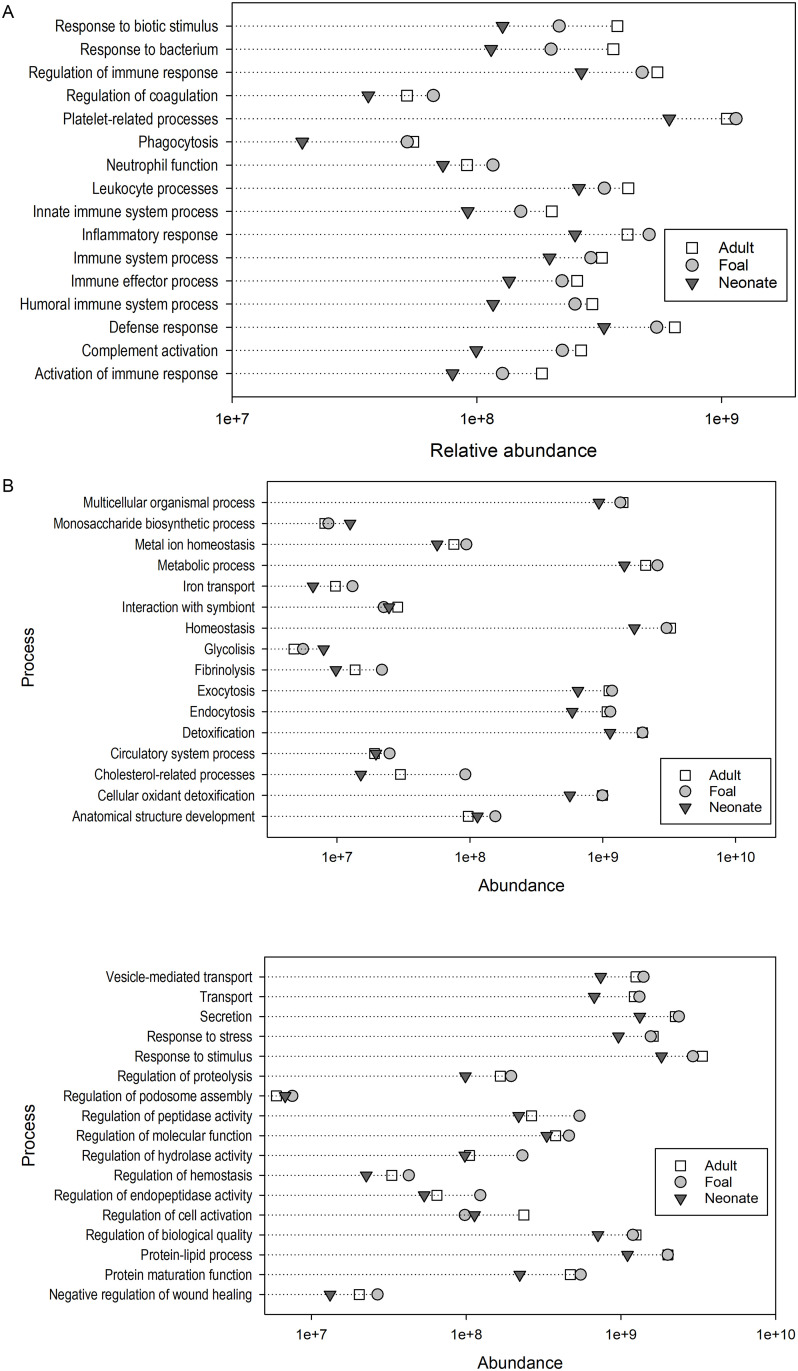
Comprehensive scatterplots of relative abundances of the biological processes identified of (A) Immune-related and (B) Non-immune related processes between neonates, foals, and adult horses. The sum of the average protein reporter ion totals for each biological process is represented in the x axes for neonates (solid triangle), foals (gray circle) and adult horses (open square). Biological processes identified inputting GO terms into GOrilla are shown in the y axes.

**Fig 3:**
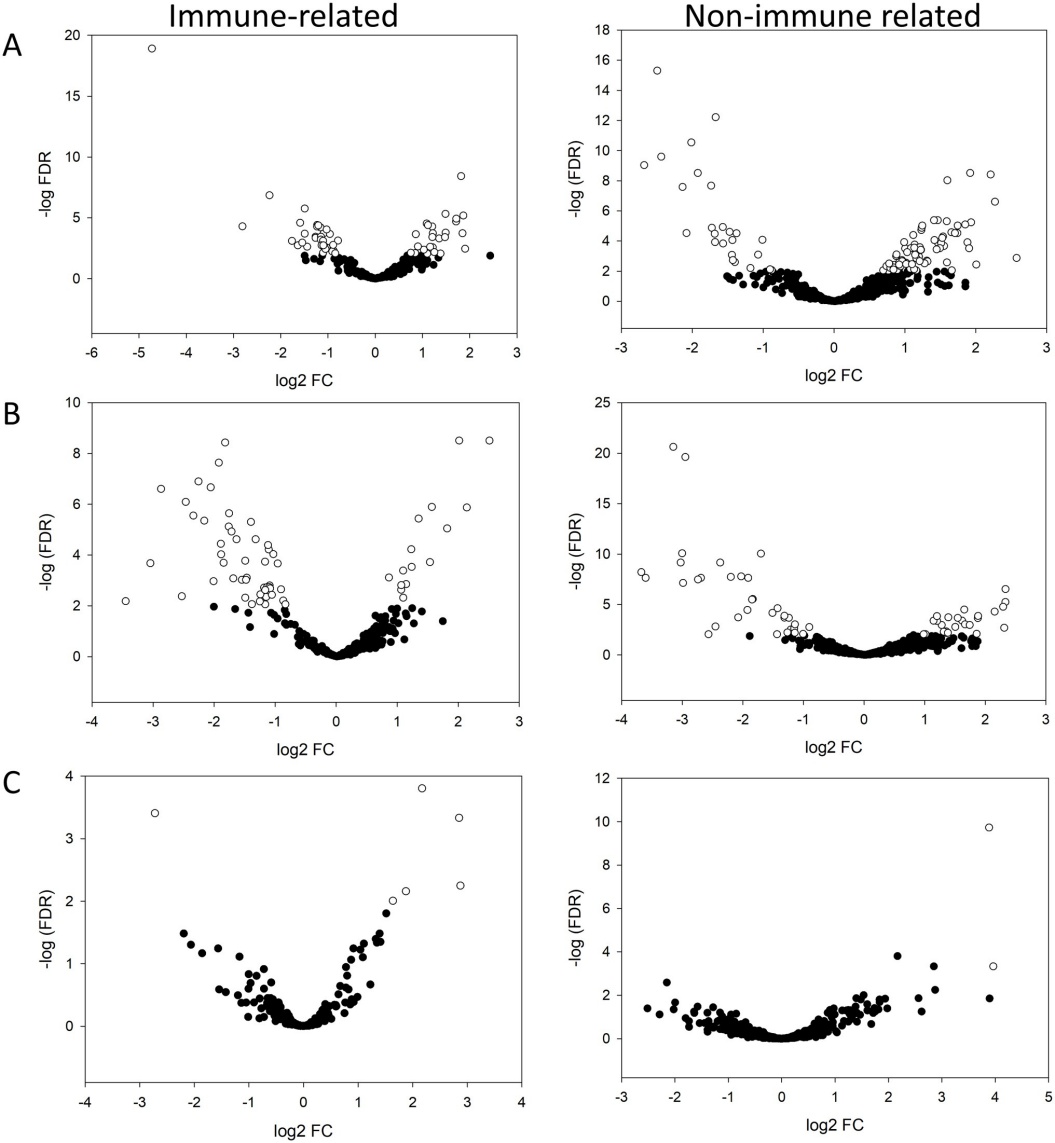
Volcano plots illustrating the protein abundance of immune-related (left panel) and non-immune related (right panel) changes between (A) Neonates vs adult horses, (B) Neonates vs foals, and (C) Foals vs adult horses. White circles represent proteins significantly different (FDR < 0.01). The log_2_ fold change is shown in the x axis. The -log of the FDR is shown in the y axis.

For these comparisons, the GO terms of all the immune-related proteins were inputted into GOrilla and grouped in 16 biological processes based on their similarity (Fig 2A). Volcano plots showing the relative protein abundance changes between groups are shown in Fig 3 and the protein candidates for each comparison are mentioned in discussion section.

#### Neonates vs adult horses

After annotation at FDR < 0.01 and a fold change > 1.80, which is the relative ratio between the two groups [30], 34 immune-related proteins were down regulated in neonates. A total of 29 immune-related proteins were up regulated in neonates (FDR < 0.01, fold change > 1.79) (Fig 2A). However, these differences in proteins did not impact the relative abundance of the immune-related processes which were all lower in neonates.

#### Neonates vs foals

Forty-seven immune-related proteins were down regulated (FDR < 0.01, fold change > 1.68) and 15 immune-related proteins were up regulated (FDR < 0.01, fold change > 2.14) in neonates (Fig 2A). The relative abundance of all the immune-related processes was lower in neonates.

#### Foals vs adult horses

Only one immune-related protein had a reduced relative abundance (FDR < 0.01, fold change = 4.45) and 5 immune-related proteins had higher relative abundance (FDR < 0.01, fold change > 3.67) in foals. This group had higher relative abundance of regulation of coagulation, inflammatory response, and platelet-related processes. Adults had higher relative abundances of the remaining immune-related processes (Fig 2A).

#### Other proteins

GO terms of the non-immune related proteins were grouped in 33 biological processes based on their process similarities using GOrilla (Fig 2B). Volcano plots showing protein relative abundance differences are presented in Fig 3.

#### Neonates vs adult horses

Twenty-four proteins were down regulated (FDR < 0.01, fold change > 1.77), and 81 proteins were up regulated (FDR < 0.01, fold change > 1.62) in neonates. Glycolysis, anatomical structure development, and monosaccharide biosynthetic processes had higher relative abundance in neonates. The rest of the non-immune related processes had higher relative abundance in adult horses (Fig 2B).

#### Neonates vs foals

Thirty-four proteins were down regulated (FDR < 0.01, fold change > 1.70) and 26 proteins were up regulated (FDR < 0.01, fold change > 1.78) in neonates. Neonates had higher relative abundances of glycolysis, interaction with symbiont, monosaccharide biosynthetic process and regulation of cell activation; foals had higher relative abundance of the other non-immune processes (Fig 2B).

#### Foals vs adult horses

Two proteins had a higher relative abundance in foals (FDR < 0.01, fold change > 2.90). Foals had higher relative abundance in anatomical structure development and iron cholesterol-related processes (Fig 2B). Both groups had similar relative abundance of multicellular organismal process, monosaccharide biosynthetic process, homeostasis, endocytosis, exocytosis, detoxification, cellular oxidant detoxification, secretion, response to stress, regulation of biological quality, and protein-lipid process. Adult horses had higher relative abundance of the remaining non-immune processes (Fig 2B).

#### Immunoglobulins

**Fig 4:**
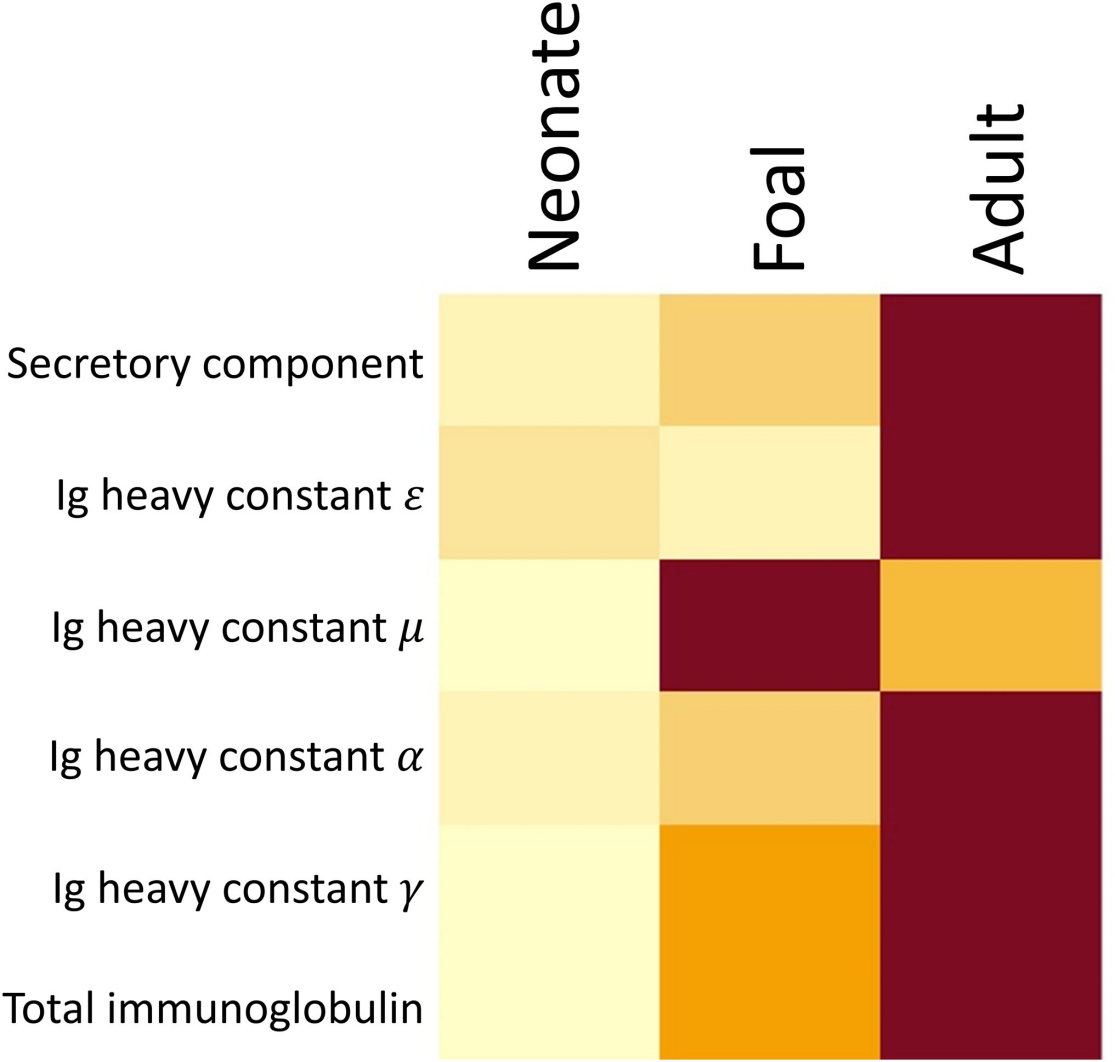
Heatmap representing the differences in total immunoglobulin and immunoglobulin heavy chain relative abundance between neonates, foals, and adult horses. Yellow tones indicate a significant lower relative abundance. Groups are shown in columns, the rows represent the immunoglobulin isotypes.

The classification of immunoglobulins into subclasses is not possible with shotgun proteomic methods in part because Uniprot does not have a deep sequence coverage of immunoglobulins [31]. Immunoglobulin isotypes can be classified based on their heavy chain [32]. Therefore, all the peptides identified as part of the same immunoglobulin isotype based on the heavy chain were grouped for comparison. Adult horses and foals presented more relative abundance of immunoglobulins. Immunoglobulin heavy constant γ was the most common peptide in all samples, followed by secretory component, immunoglobulin heavy constant α, immunoglobulin heavy constant μ and in last place immunoglobulin heavy constant ε. The relative abundances were significantly different between groups. Immunoglobulin heavy constant γ, immunoglobulin heavy constant α, immunoglobulin heavy constant μ and secretory component had lower relative abundance in neonates (FDR < 0.01) and there was no difference between foals and adult horses (FDR > 0.05). Immunoglobulin heavy constant ε had lower relative abundance in neonates and foals than in adult horses (FDR < 0.01), Fig 4.

## Discussion

The present study describes a marked variation between the BALF proteome of neonate horses and that of foals and adult horses. To the best of the authors’ knowledge, this is the first time the proteomics of equine neonates and foals BALF is evaluated. Differences in proteome by age was previously reported using not only the TMT method [33], but also others such as two-dimensional difference gel electrophoresis [34, 35]. Profile differences were assessed after ortholog mapping of the identified peptides in all the individuals with BLAST alignment sequence similarity between horse and human proteins. This methodology is commonly used for species that have less developed database annotations [36, 37]. The authors acknowledge that while protein names have several regularities, some can differ among different species [38].

A total of 704 proteins were successfully classified based on their molecular function and biological process. The most frequent molecular functions identified were binding and catalytic activity and the most common biological processes were cellular process, metabolic process, and biological regulation. This is consistent with that described for adult healthy horses, non-smoking humans, mice, and newborn pigs [17, 39–41].

For the group comparisons, the biological processes were identified using GO terms and GOrilla. This resulted in 101 biological processes that were combined into 49 processes (16 immune-related and 33 non-immune related) based on their similarity. Neonates had a reduced relative abundance of most processes when compared to foals and to adult horses.

Neonates had less relative abundance of immune-related proteins than the other two groups. Circulating cytokines and complement proteins have been shown to be lower in neonates than in adult horses [42, 43]. In the present study, the relative abundance of complement proteins in the BALF, which are key for microbial opsonization [44], was lower in neonate than in foals and adult horses. Specifically, C2a, C4b-binding protein, properdin, C1q subunits B and C, C4, and C8 were lower in neonates. C1q is deemed essential for opsonization and killing of *R*. *equi* [45]. Thus, this difference in complement composition and its role in age-associated susceptibility to *R*. *equi* warrants further investigation. Other immune-related proteins that had a lower relative abundance in BALF of neonates were macrophage receptor with collagenous structure (MARCO), bactericidal/permeability increasing (BPI) fold-containing family B member 1 protein (BPIFB1), and ferritin. Macrophage receptor MARCO mediates binding and ingestion of unopsonized environmental particles and pathogens including Gram-negative and Gram-positive bacteria [46, 47]. BPFB1 belongs to a group of innate immune proteins that exerts its antimicrobial effect binding to lipopolysaccharides of Gram-negative bacteria present in the respiratory tract [48]. This protein is abundant in nasopharyngeal and respiratory secretions of fetal and adult humans [49]. Ferritin is essential for iron storage and iron availability needed for cellular activities [50]. In horses and humans, ferritin is part of the acute phase response increasing as a result of bacterial and viral infection and inflammation [51–53]. This lower abundance of ferritin may result in higher availability of iron for pathogens.

When compared to adult horses, neonates had relatively lower abundance of lipopolysaccharide-binding protein (LBP), a modulator that enables rapid response against LPS of Gram-negative bacteria in mice [54] among other immune-related proteins (S3 Table). Factor H, C6 and C9, all part of the complement cascade [44] and macrophage colony stimulator factor, a protein that establishes and maintains tissue-resident macrophages [55] had lower relative abundance in neonates than in foals (Fig 2A, S3 Table).

Neonates had the highest relative abundance of mucin-1 and lysozyme C. Mucin-1 is a transmembrane glycoprotein that along with other mucins, provides a protective barrier against pathogens [56, 57]. However, this glycoprotein also plays an anti-inflammatory role following the activation of host inflammation in response to a variety of infectious insults [58]. Whether this is also the case in equine neonates with respiratory infection remains to be determined. Lysozyme C is an antimicrobial protein that can hydrolyze the β-1,4-glycosidic bond present in the polysaccharide layer of Gram-positive bacteria cell walls [59]. Similar to what is reported here, this protein decreases with age in humans [60].

Surfactant proteins have a role in lung protection. This group of proteins reduces the surface tension at the air-liquid interface of the lung and modulates the inflammatory response against pathogens [15]. In humans, surfactant protein A is higher in children than in adults [61]. Similar differences were not observed in our study, but this may be due to the low number of individuals included.

Foals and adult horses had similar immune-related protein abundance (Fig 2A). Five proteins were relatively more abundant in foals including apolipoprotein AI which is part of the apolipoproteins family and has been identified in human BALF [62]. This high-density lipoprotein prevents viral penetration, facilitates complement-mediating bacterial killing, and provides protection against parasites [63]. Apolipoprotein I expression was found higher in adult humans that in children [64]. Ferritin was the only protein with a higher relative expression in adult horses, and has also been shown to increase with age in BALF of rats [34].

Non-immune related proteins such as peroxiredoxin-6, aquaporin-1 and ezrin, all of them related to lung development and metabolism, had a higher relative abundance in neonates than in the other groups. Similar age differences have been reported in humans [65–67]. Foals and adult horses had similar protein expression levels of non-immune related proteins. Only superoxide dismutase and beta-galactosidase were higher in foals than in adult horses.

Immunoglobulin G is the most abundant Ig in BALF of adult horses, followed by IgA, IgE and IgM [68]. If all the peptides grouped based on the heavy chain are part of each specific immunoglobulin isotype [32], the Ig abundances in the BALF of neonates and foals in this study followed a similar pattern of abundances. As expected, neonates had the lowest relative abundance of all the immunoglobulins, as production of this immunoglobulin does not reach adult levels until 5–8 weeks of life [69] and mare’s placentation prevents in utero immunoglobulin transfer [1, 12].

This study is not without limitations. Only a small number of horses was included in each group and all the adult horses were females. All horses were Thoroughbreds and were housed in the same environment. Additional work is needed before this information can be extrapolated to other breeds. The TMT labeling is an isobaric method that allows the analysis of peptides and infers protein ID based on unique peptides. This method is followed by IRS normalization, that removes compositional bias, ensuring fully confidentiality when comparing proteins across groups with equal variances [21, 29]. However, as in most of quantitative proteomic methods, the results are expressed as relative abundance changes instead of absolute quantification. Other techniques such as enzyme-linked immunosorbent assay and immunoblotting analysis are needed to confirm the single protein differences observed in our study. An additional limitation is the use of algorithms from open-access tools to predict the protein function which may not be completely accurate.

Overall, equine neonates had lower relative abundance of immune-related proteins than foals and adult horses. In particular, those associated with antibacterial function such as complement proteins, macrophage receptor MARCO, BPI fold-containing family B member 1, ferritin, and immunoglobulins. The lower relative abundance of immune-related proteins in BALF of neonates may contribute to their susceptibility to pulmonary infections. Our study provides the bases for further evaluation of the role some of these single proteins may have in the protection of the neonatal lung.

## Supporting information

S1 Fig Scatterplots of protein intensities from the two pool channels from each of the four TMT experiments. (A) Pooled standards before IRS. (B) Pooled standards after IRS normalization process.
(DOCX)

S2 Fig Intensity distributions for all 17 samples after TMM normalization. The X axis represents the samples and Y axis represent the log of the intensity. Foals are represented in red, adult horses in green, and neonates in blue.
(TIF)

S1 Table Proteins present in all neonate samples and their protein expression levels (PEI). (A) Uniprot identification, molecular function, and biological processes are included. (B) Fold change of immune related proteins of neonates compared to adults. (C) Fold change of non-immune related proteins of neonates compared to adults.
(XLSX)

S2 Table Subclassification and percentages of the proteins involved in the immune system process. Proteins not recognized by GOrilla but involved in immunity were added to the “Immune system process” section. Uniprot accession numbers are included.
(DOCX)

S3 Table Heatmap representing the differences of protein expression levels of (A) proteins involved in the immune system process and (B) proteins involved in other biological processes (FDR < 0.01). The maroon and orange colors indicate higher expression levels. Yellow color indicates a significant lower expression. Each column represents the group of horses (neonate, foal, and adult) and rows represent the proteins.
(DOCX)
