# Correction: Comparison of the broncoalveolar lavage fluid proteomics between foals and adult horses

**DOI:** 10.1371/journal.pone.0306211

**Published:** 2024-06-21

**Authors:** Alejandra A. Rivolta, Adina R. Bujold, Phillip A. Wilmarth, Brett S. Phinney, Joseph P. Navelski, David W. Horohov, Macarena G. Sanz

The word "Bronchoalveolar" is misspelled in the article title. The correct title is: Comparison of the bronchoalveolar lavage fluid proteomics between foals and adult horses. The correct citation is: Rivolta AA, Bujold AR, Wilmarth PA, Phinney BS, Navelski JP, Horohov DW, et al. (2023) Comparison of the bronchoalveolar lavage fluid proteomics between foals and adult horses. PLoS ONE 18(9): e0290778. https://doi.org/10.1371/journal.pone.0290778.
