# Supplementary figures and images for: Comparison of the broncoalveolar lavage fluid proteomics between foals and adult horses
Source: PLoS One. 2023 Sep 5;18(9):e0290778. doi: 10.1371/journal.pone.0290778 (PMC10479908; doi:10.1371/journal.pone.0290778)

S1a


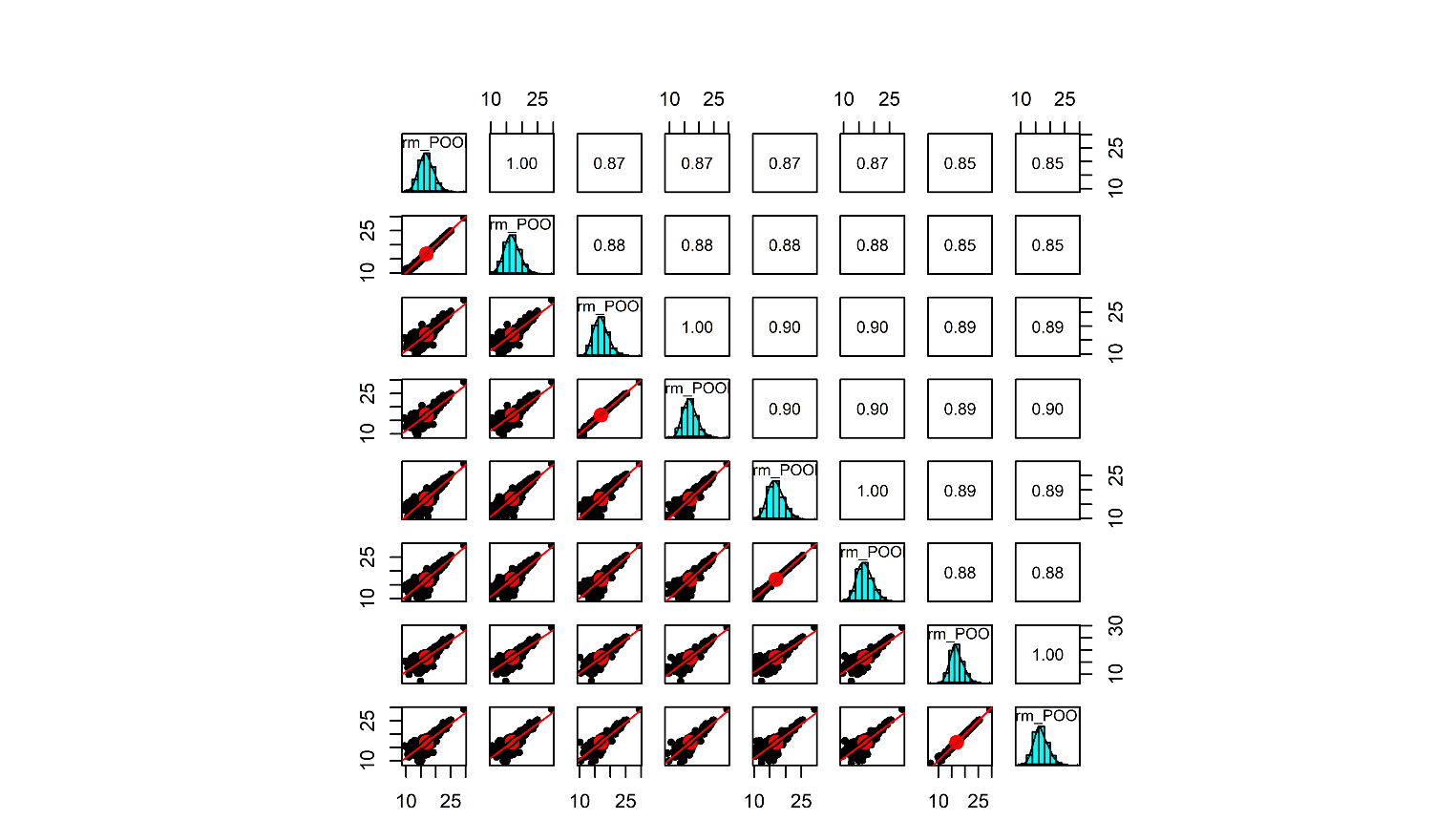


S1b


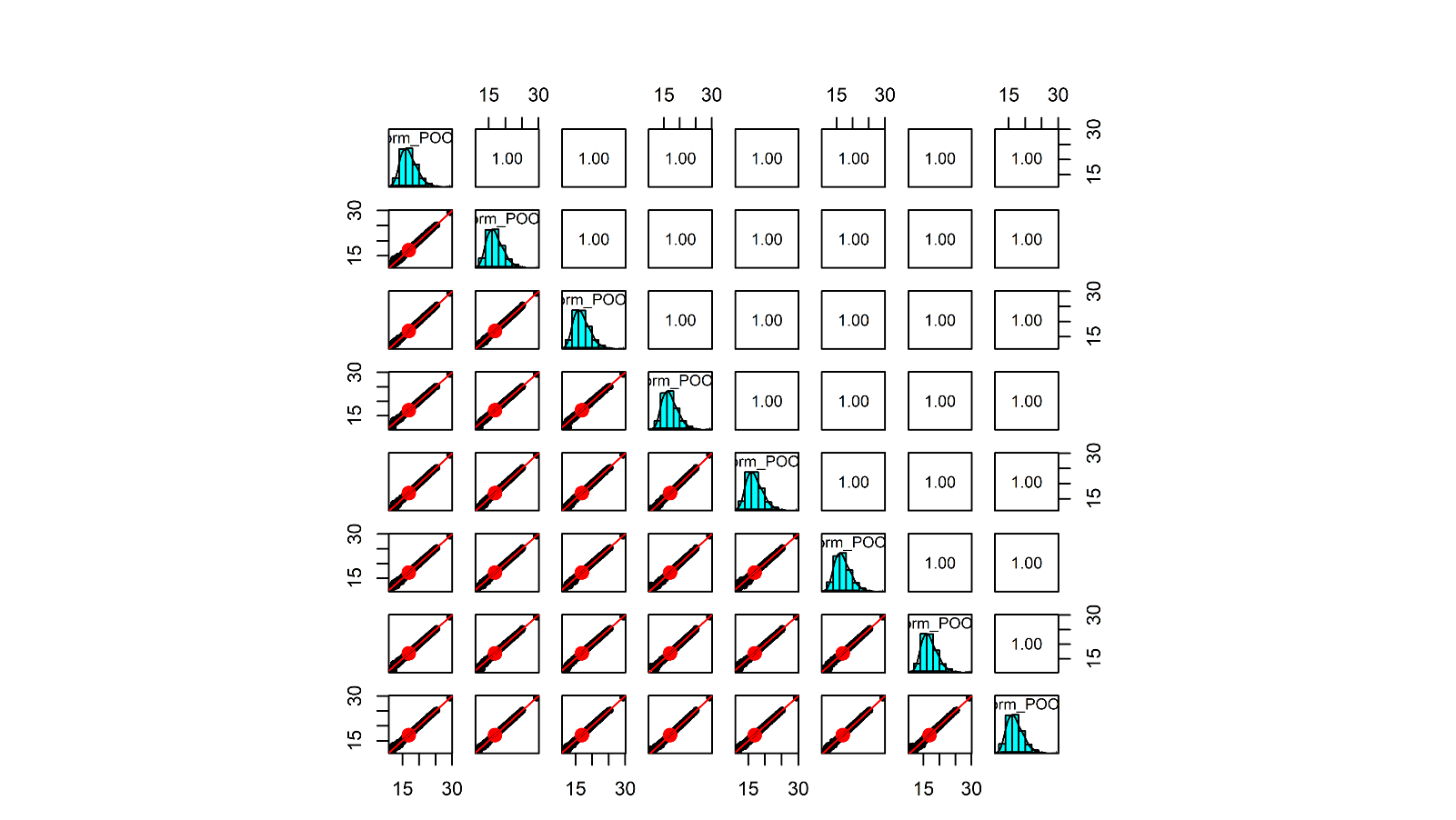

Supplement: S1 Fig — (A) Pooled standards before IRS. (B) Pooled standards after IRS normalization process. (DOCX) [file pone.0290778.s001.docx]

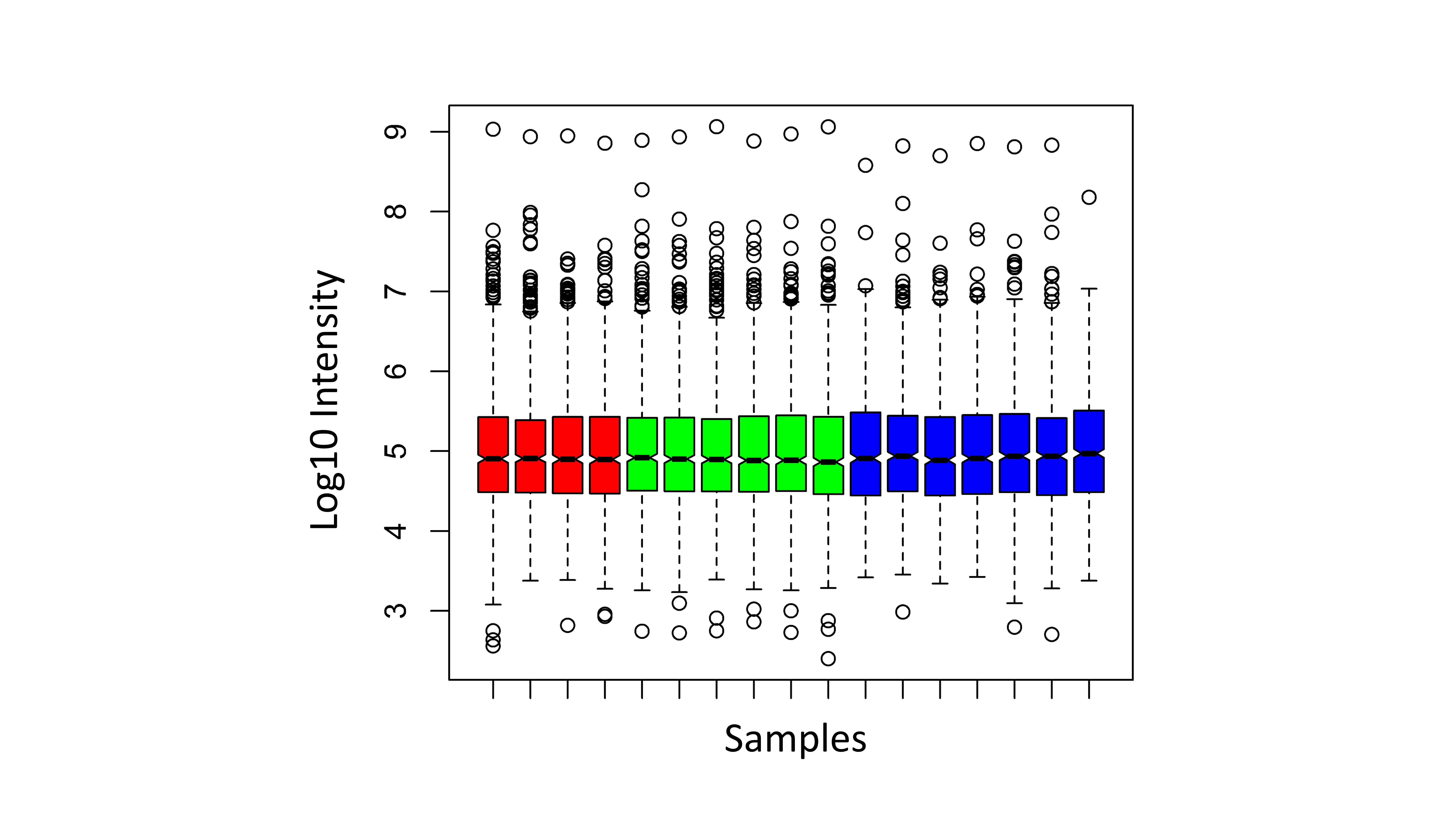

Supplement: S2 Fig — The X axis represents the samples and Y axis represent the log of the intensity. Foals are represented in red, adult horses in green, and neonates in blue. (TIF) [file pone.0290778.s002.tif]

1. Heatmap immune-related proteins


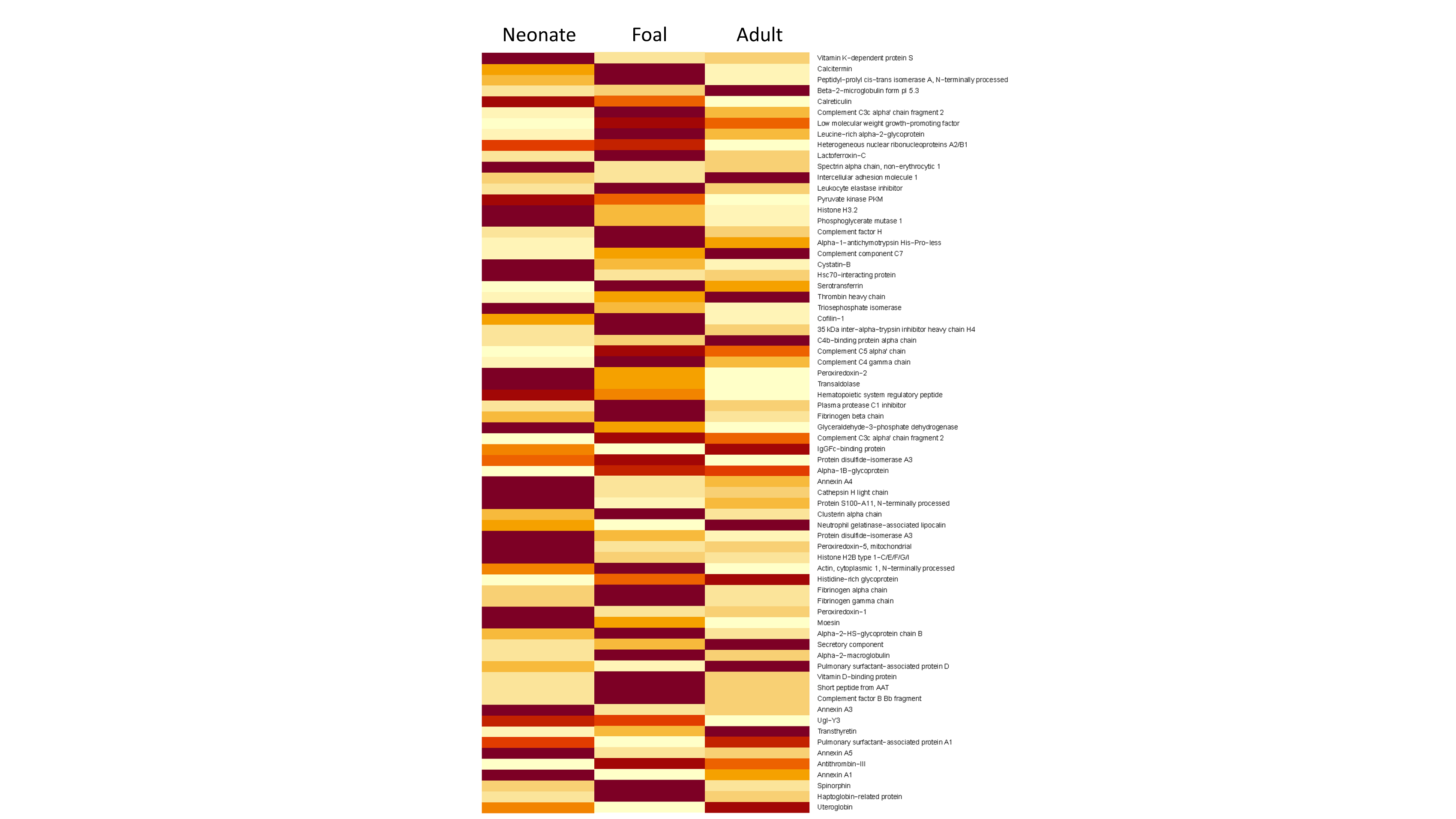


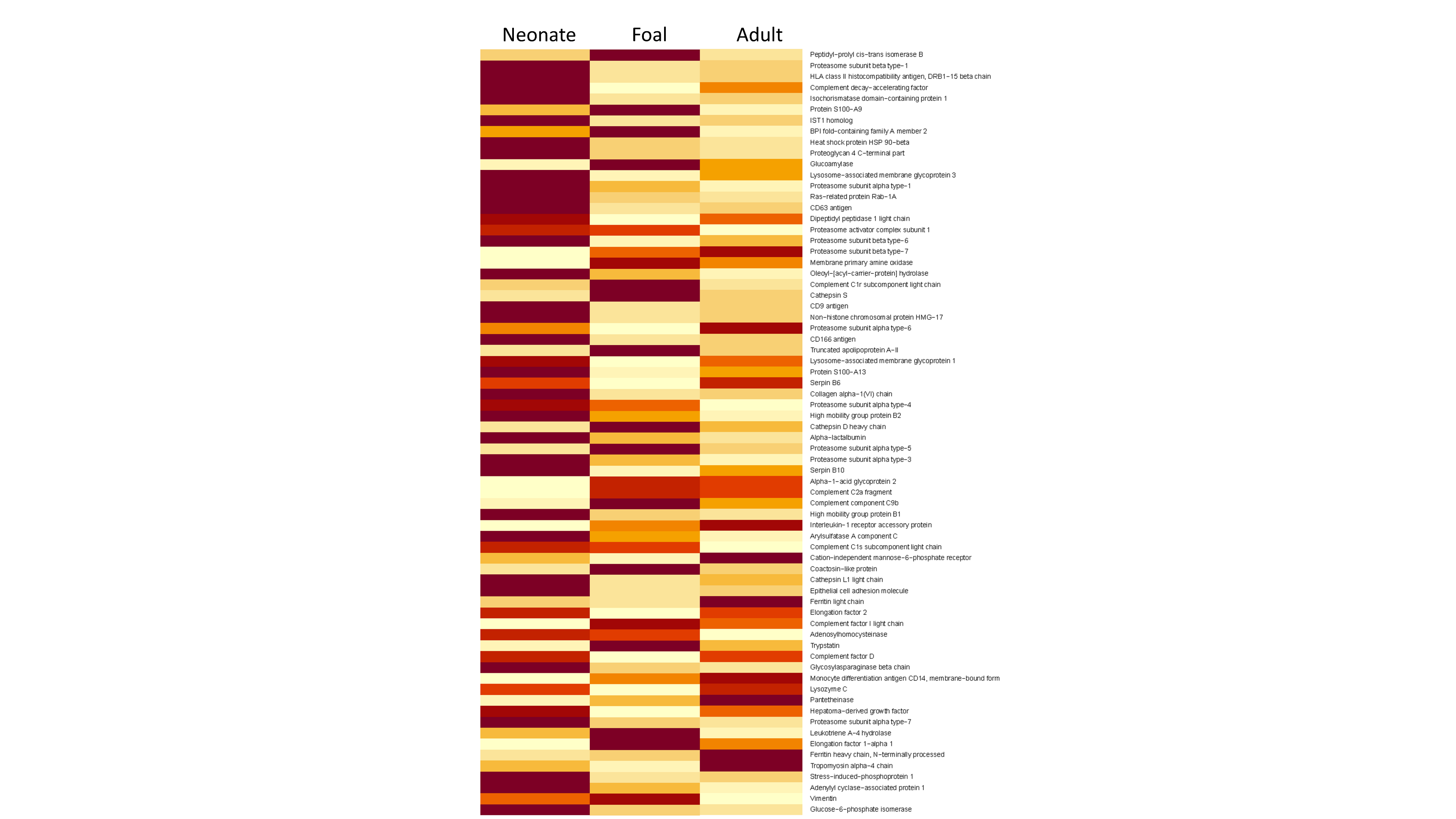


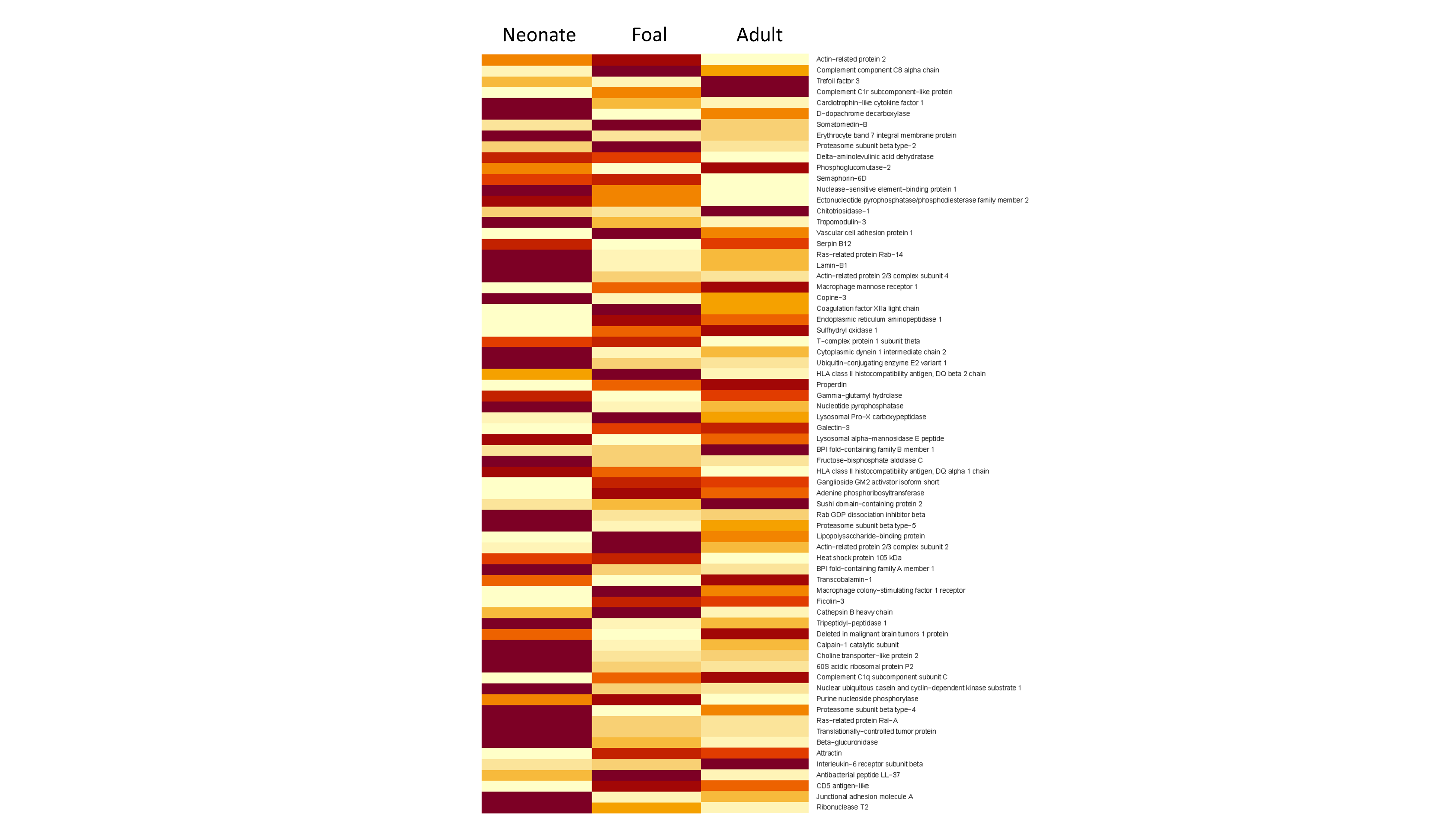


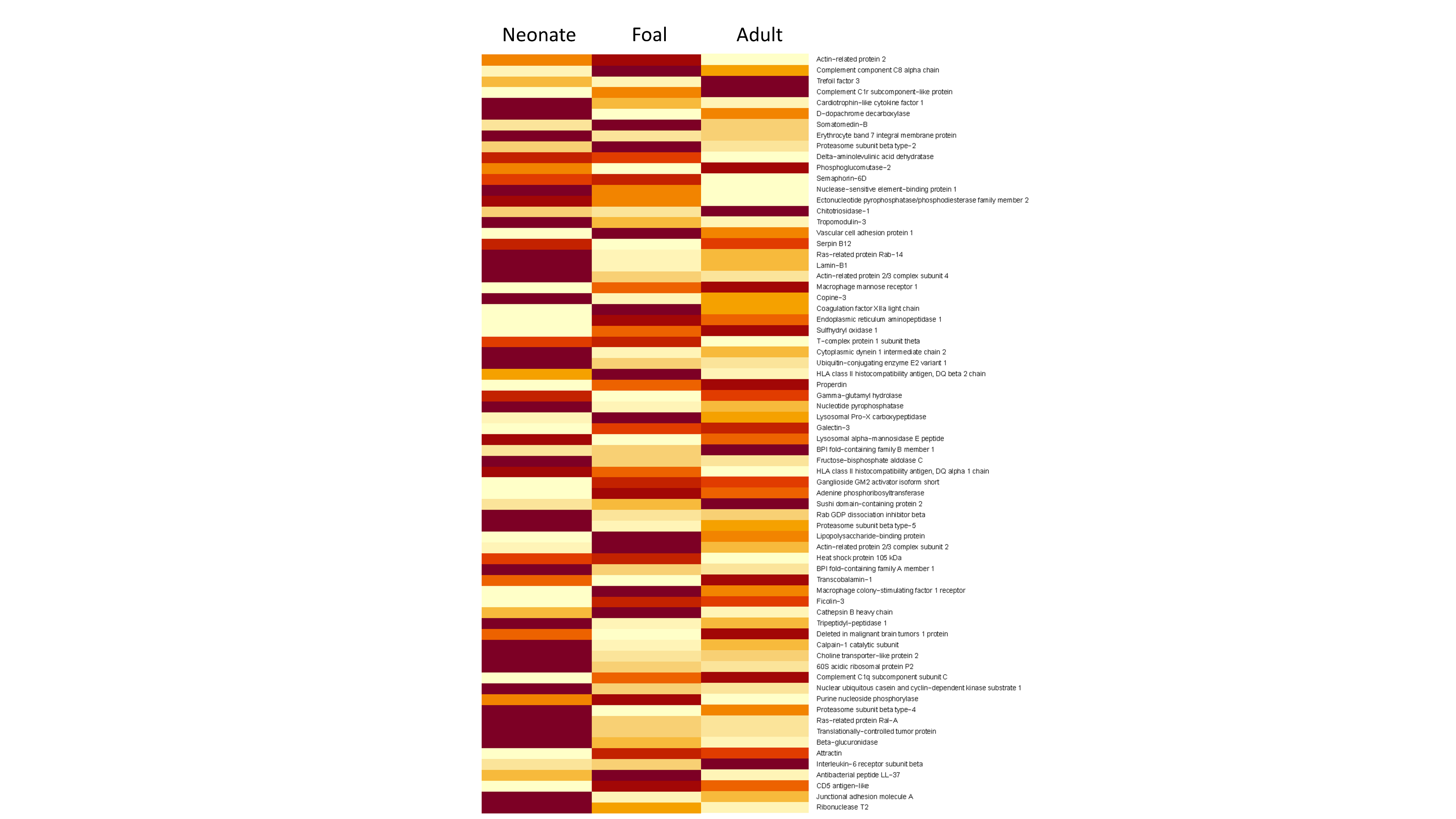


1. Heatmap other proteins present in BALF


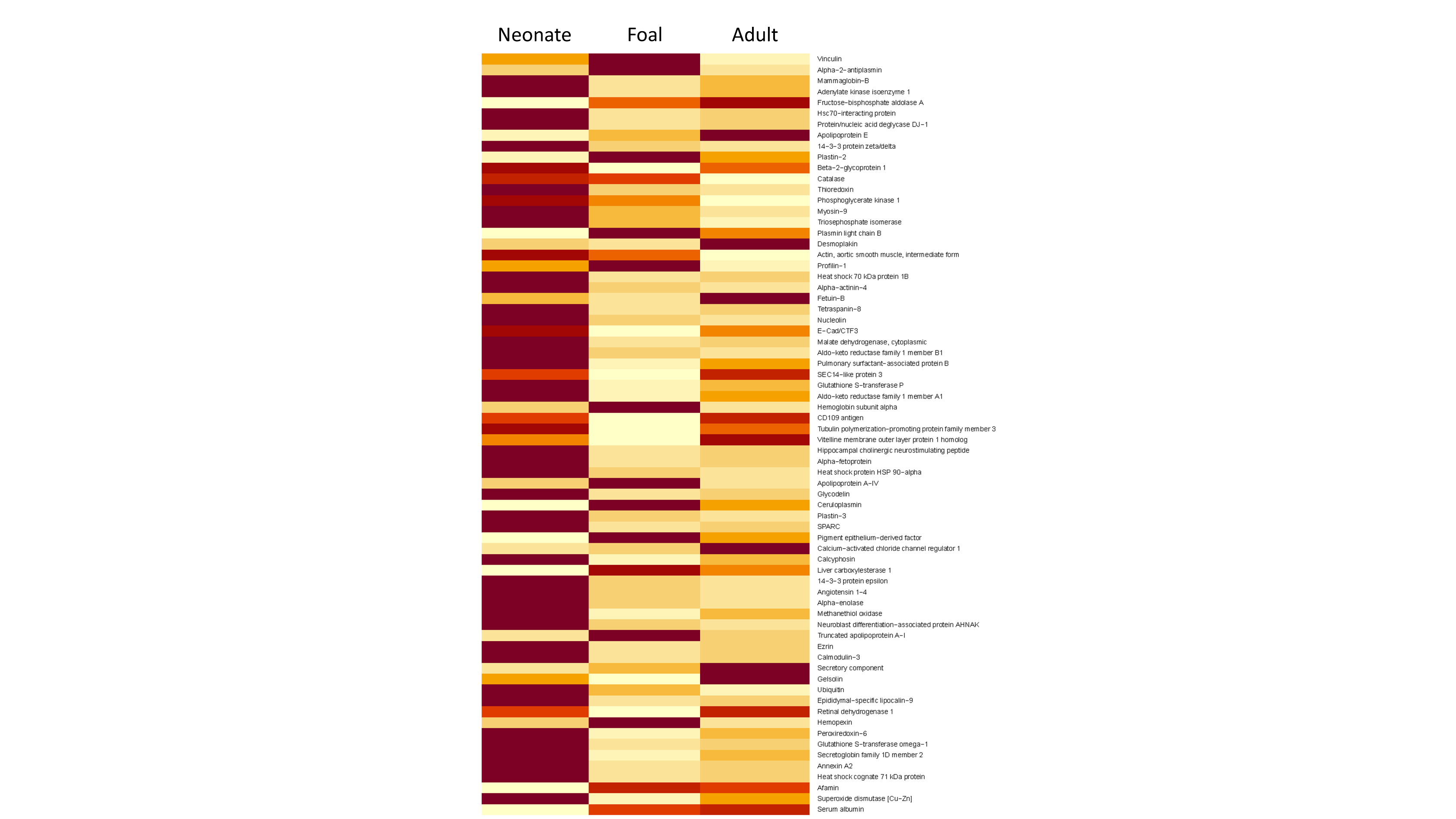


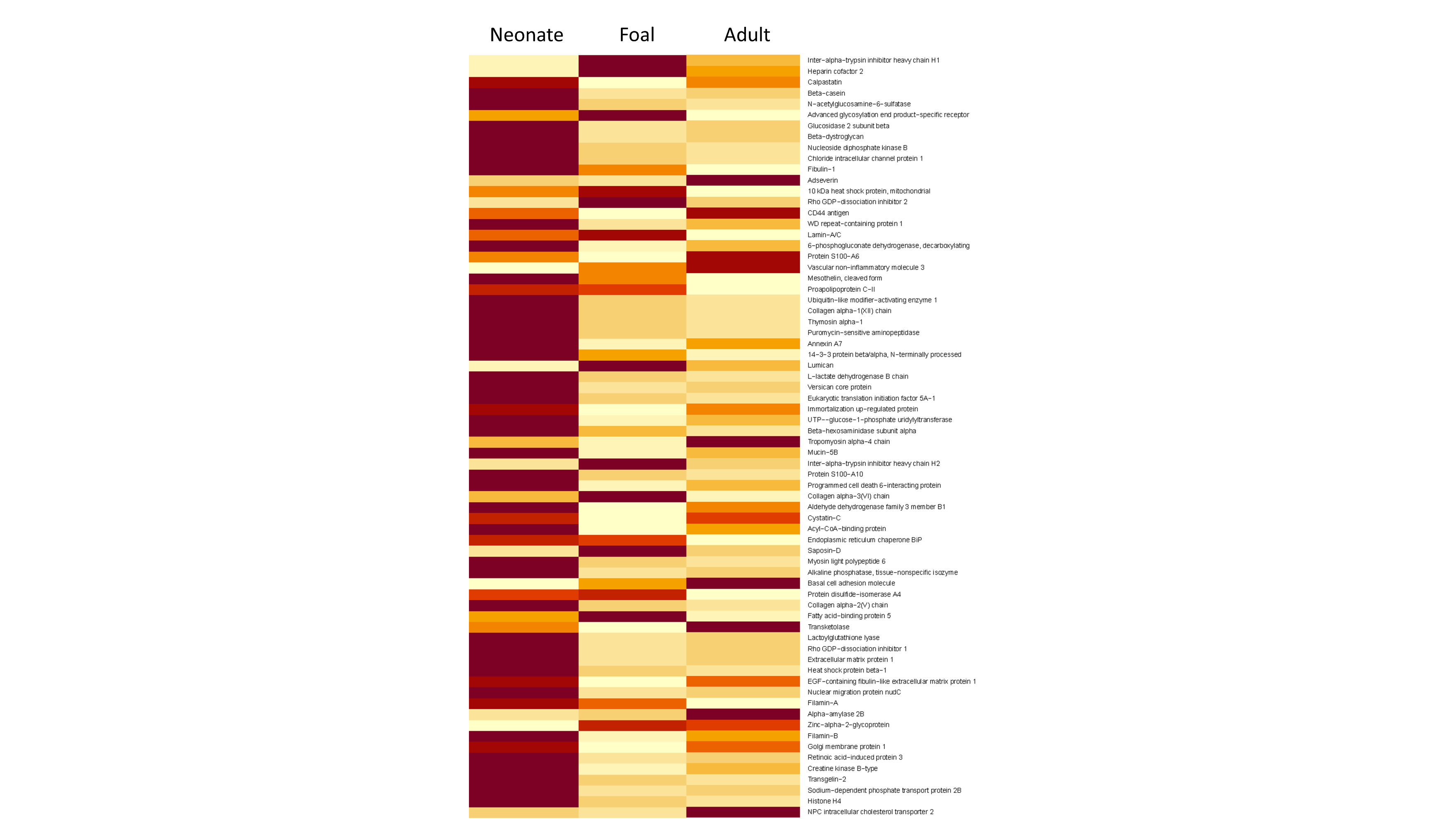


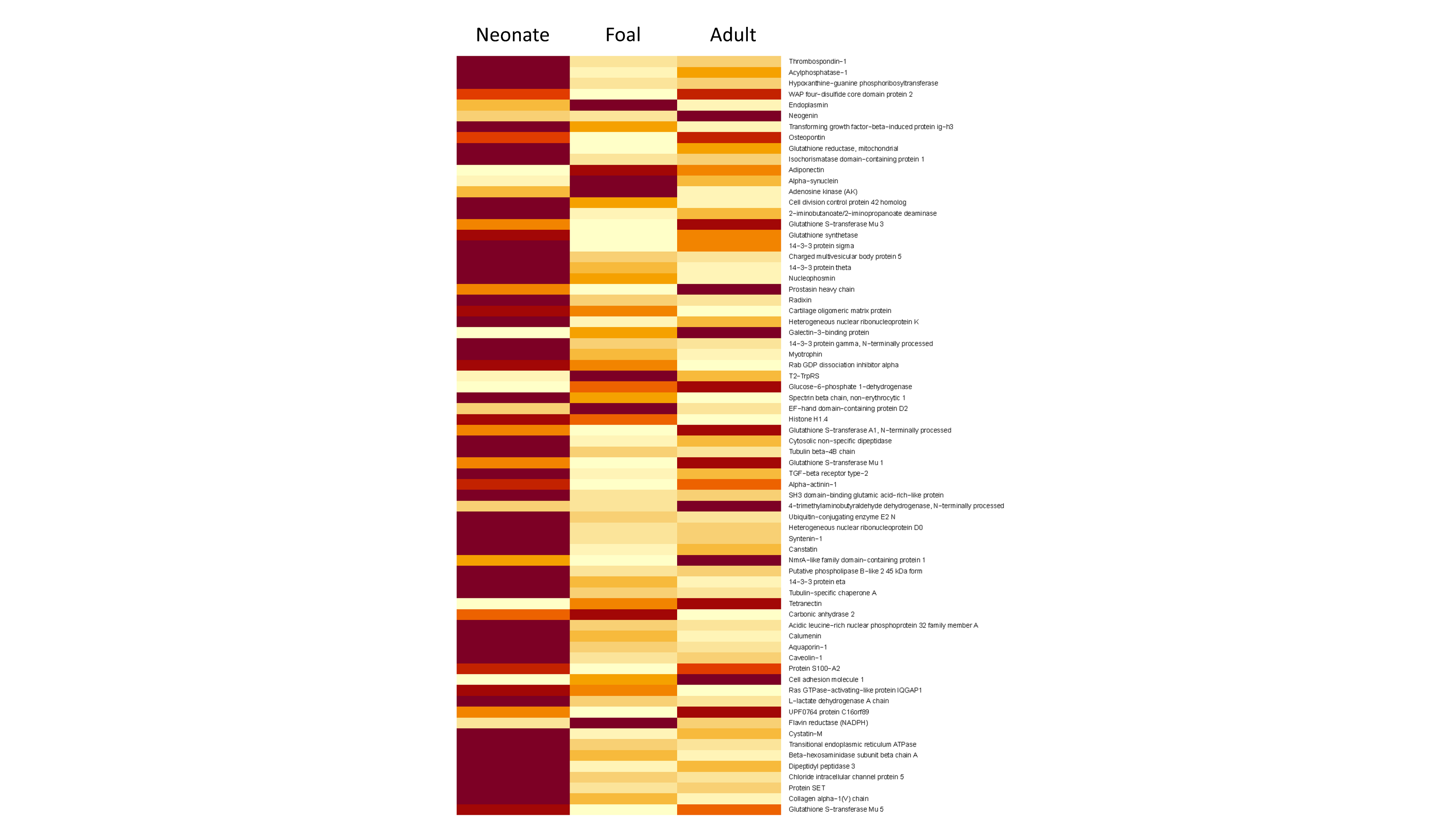


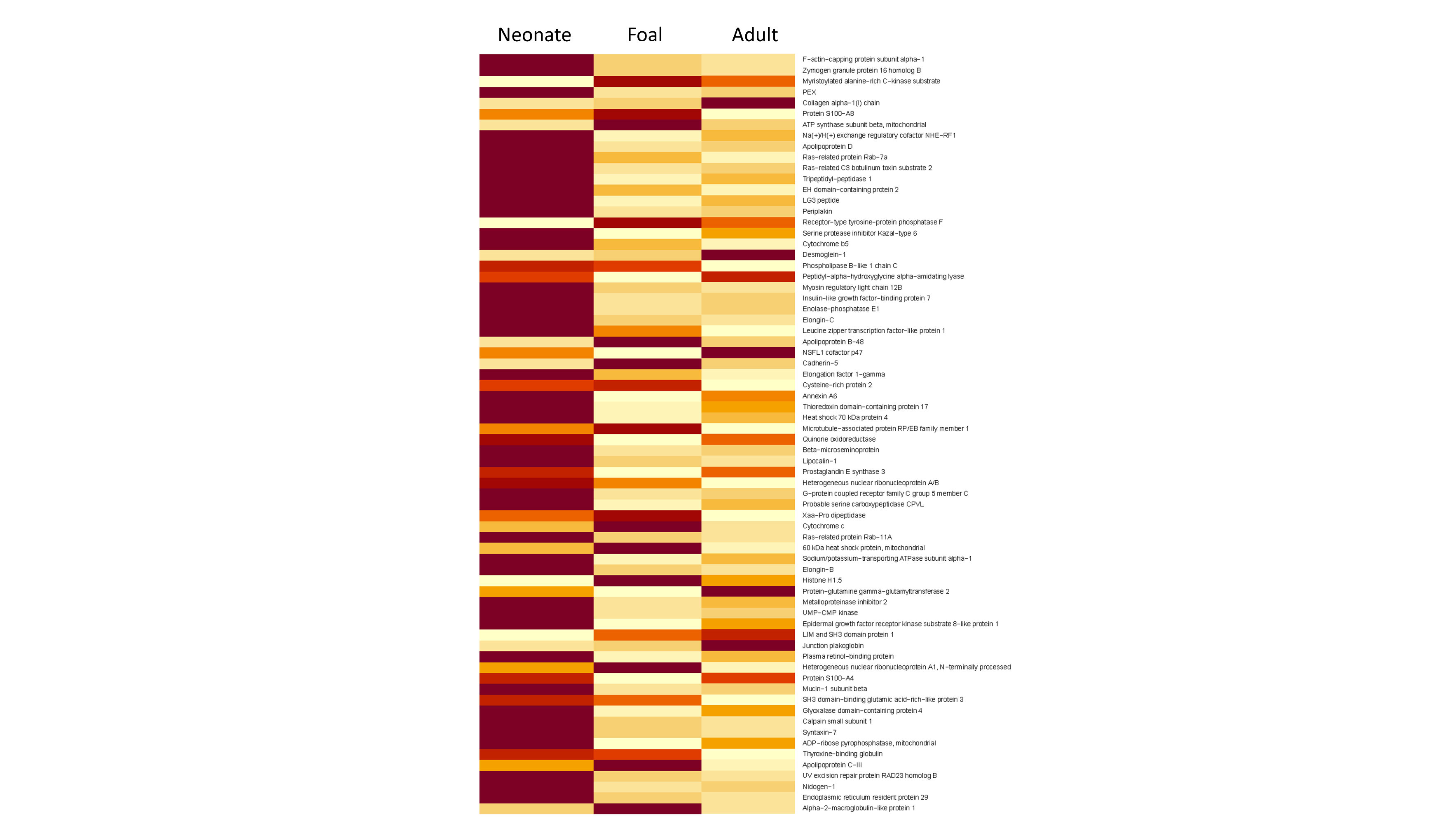


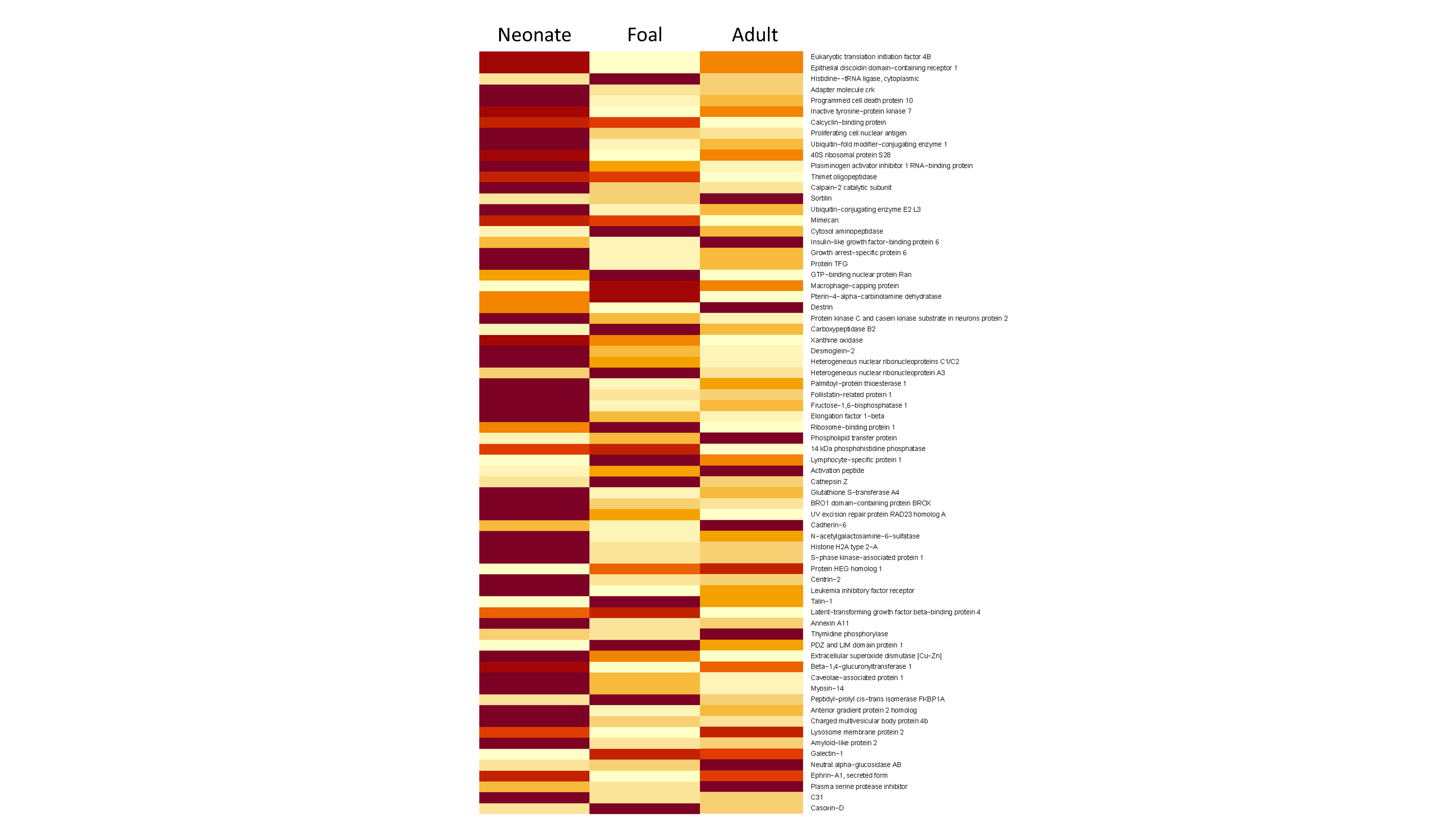


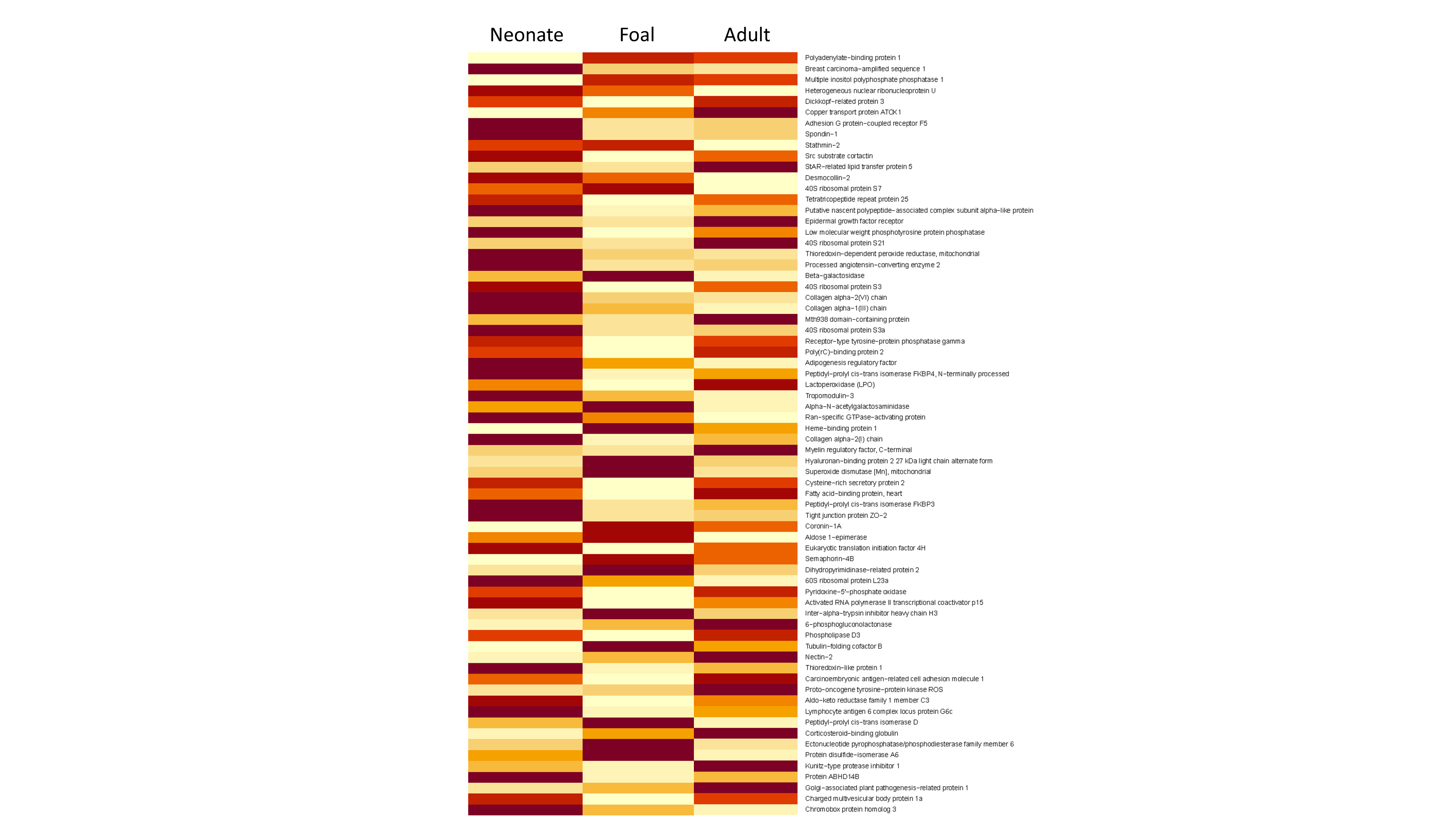


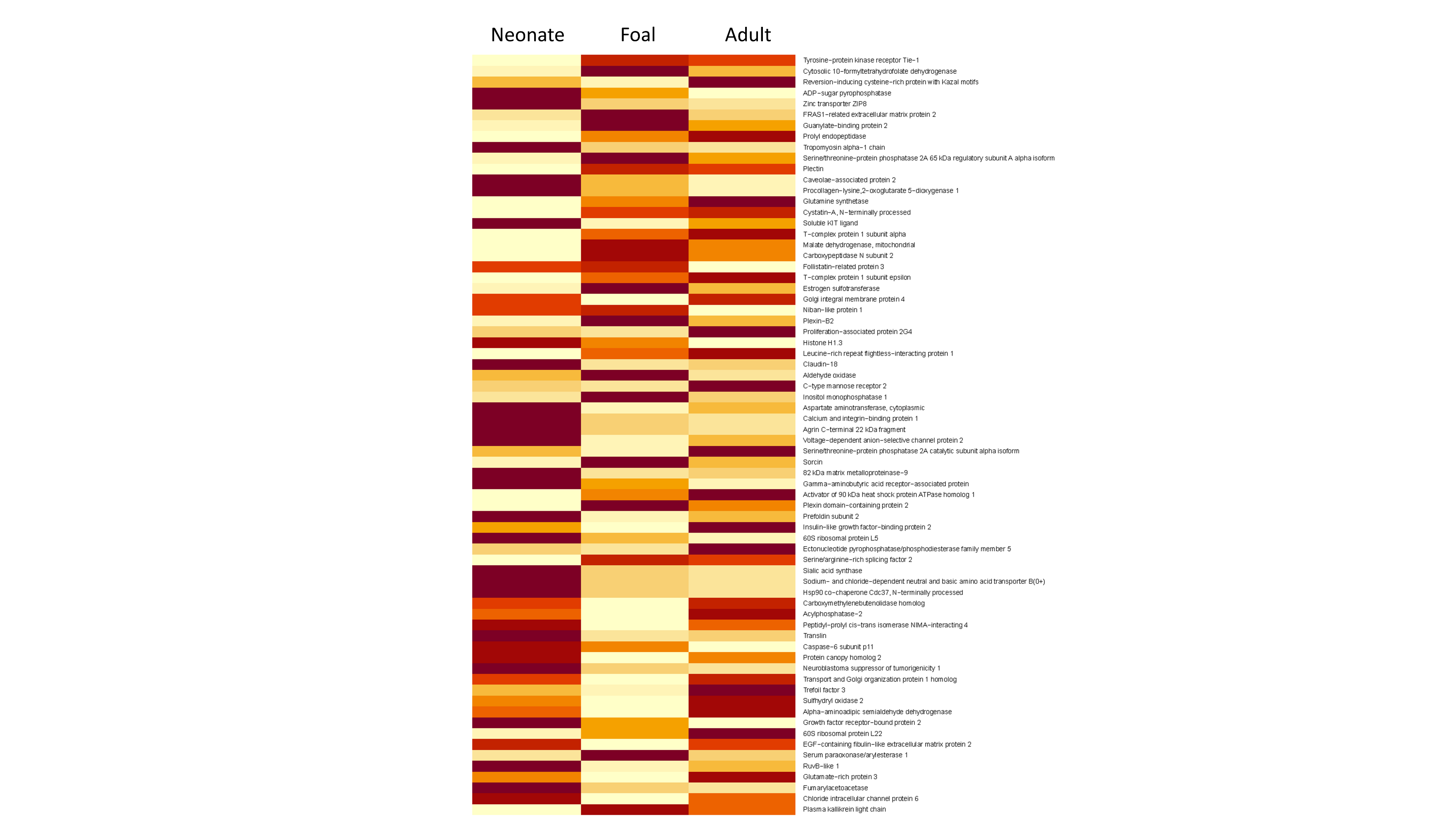


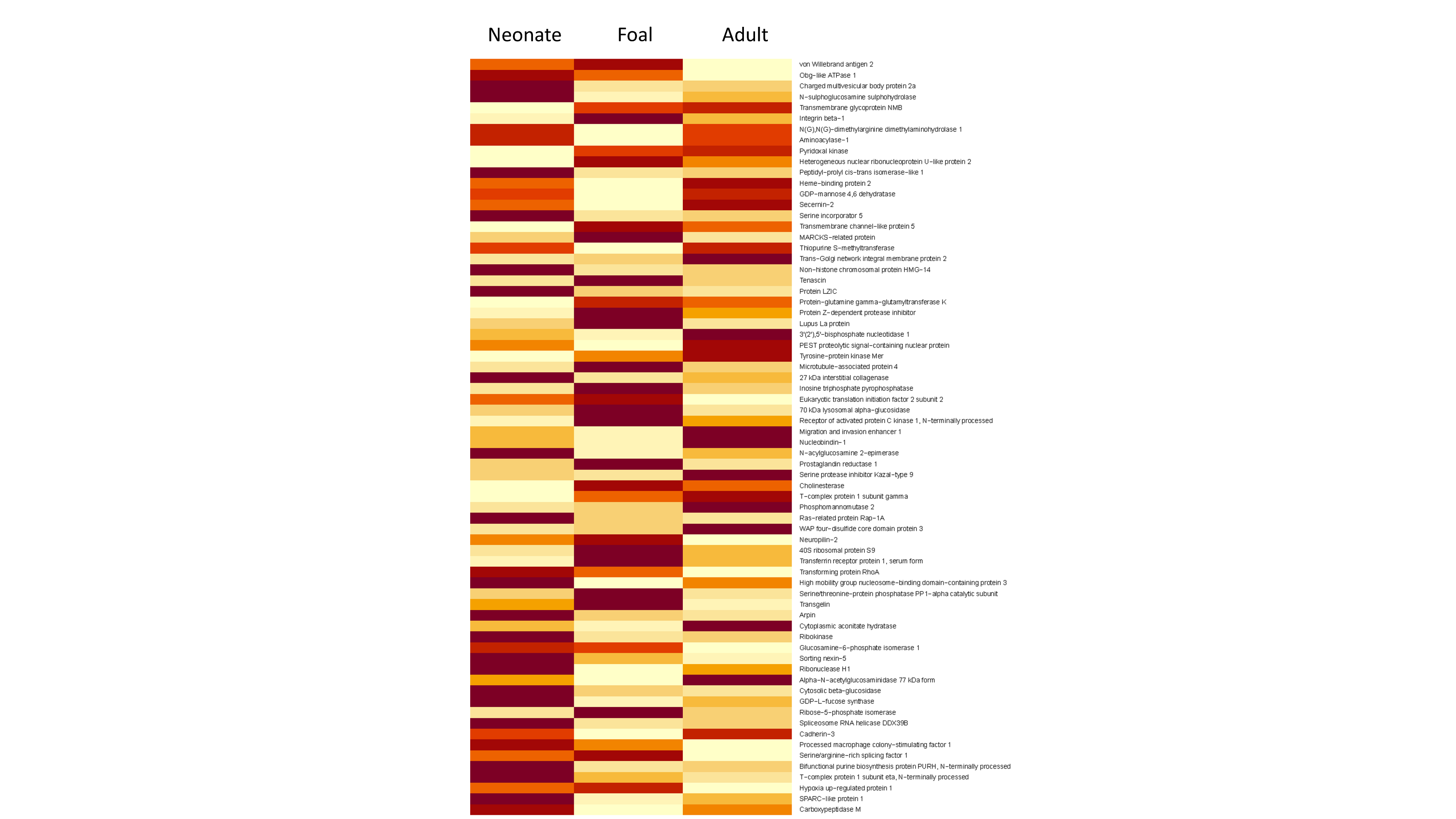


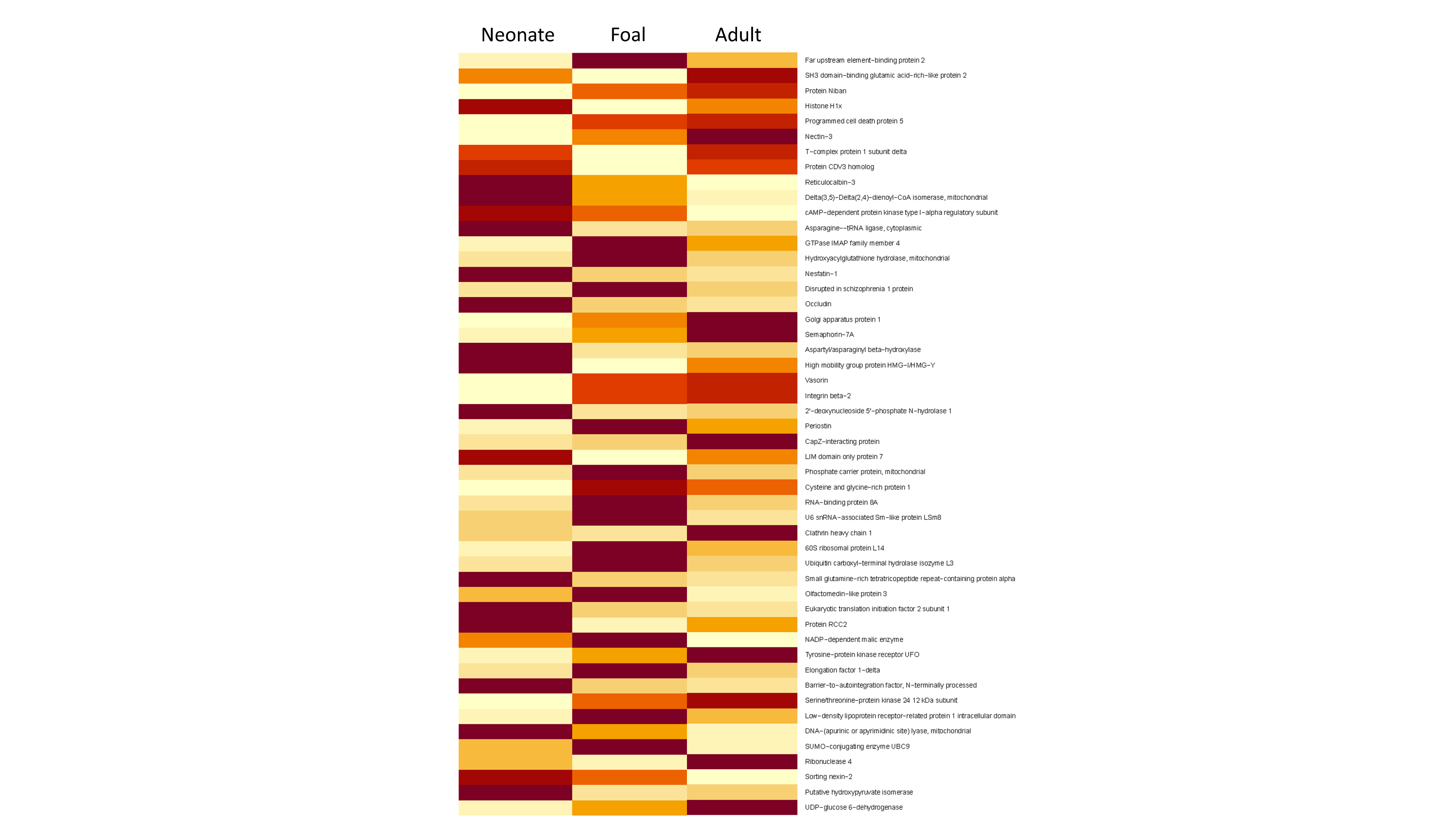

Supplement: S3 Table — The maroon and orange colors indicate higher expression levels. Yellow color indicates a significant lower expression. Each column represents the group of horses (neonate, foal, and adult) and rows represent the proteins. (DOCX) [file pone.0290778.s005.docx]
