# Supplementary material for: Comparison of the broncoalveolar lavage fluid proteomics between foals and adult horses
Source: PLoS One. 2023 Sep 5;18(9):e0290778. doi: 10.1371/journal.pone.0290778 (PMC10479908; doi:10.1371/journal.pone.0290778)
Supplement: S2 Table — Proteins not recognized by GOrilla but involved in immunity were added to the “Immune system process” section. Uniprot accession numbers are included. (DOCX) [file pone.0290778.s004.docx]

| **Description** | **Accession** | **Name** |
| --- | --- | --- |
| **Immune system process (GO:0002376) 44.3%** | [P23526](http://www.uniprot.org/uniprot/P23526) | Adenosylhomocysteinase |
|  | [Q96DR5](http://www.uniprot.org/uniprot/Q96DR5) | BPI fold-containing family A member 2 |
|  | [P27797](http://www.uniprot.org/uniprot/P27797) | Calreticulin |
|  | [Q13740](http://www.uniprot.org/uniprot/Q13740) | CD166 antigen |
|  | [P02452](http://www.uniprot.org/uniprot/P02452) | Collagen alpha-1(I) chain |
|  | [P16422](http://www.uniprot.org/uniprot/P16422) | Epithelial cell adhesion molecule |
|  | [Q06830](http://www.uniprot.org/uniprot/Q06830) | Peroxiredoxin-1 |
|  | [P32119](http://www.uniprot.org/uniprot/P32119) | Peroxiredoxin-2 |
|  | [P13796](http://www.uniprot.org/uniprot/P13796) | Plastin-2 |
|  | [P30101](http://www.uniprot.org/uniprot/P30101) | Protein disulfide-isomerase A3 |
|  | [O00233](http://www.uniprot.org/uniprot/O00233) | 26S proteasome non-ATPase regulatory subunit 9 |
|  | [Q13442](http://www.uniprot.org/uniprot/Q13442) | 28 kDa heat- and acid-stable phosphoprotein |
|  | [P05387](http://www.uniprot.org/uniprot/P05387) | 60S acidic ribosomal protein P2 |
|  | [P61160](http://www.uniprot.org/uniprot/P61160) | Actin-related protein 2 |
|  | [O15144](http://www.uniprot.org/uniprot/O15144) | Actin-related protein 2/3 complex subunit 2 |
|  | [P59998](http://www.uniprot.org/uniprot/P59998) | Actin-related protein 2/3 complex subunit 4 |
|  | [P61158](http://www.uniprot.org/uniprot/P61158) | Actin-related protein 3 |
|  | [P07741](http://www.uniprot.org/uniprot/P07741) | Adenine phosphoribosyltransferase |
|  | [P00709](http://www.uniprot.org/uniprot/P00709) | Alpha-lactalbumin |
|  | [P15144](http://www.uniprot.org/uniprot/P15144) | Aminopeptidase N |
|  | [P09525](http://www.uniprot.org/uniprot/P09525) | Annexin A4 |
|  | [Q9ULZ3](http://www.uniprot.org/uniprot/Q9ULZ3) | Apoptosis-associated speck-like protein containing a CARD |
|  | [P15289](http://www.uniprot.org/uniprot/P15289) | Arylsulfatase A component C |
|  | [P08236](http://www.uniprot.org/uniprot/P08236) | Beta-glucuronidase |
|  | [Q9NP55](http://www.uniprot.org/uniprot/Q9NP55) | BPI fold-containing family A member 1 |
|  | [Q9UQB8](http://www.uniprot.org/uniprot/Q9UQB8) | Brain-specific angiogenesis inhibitor 1-associated protein 2 |
|  | [P07384](http://www.uniprot.org/uniprot/P07384) | Calpain-1 catalytic subunit |
|  | [P00915](http://www.uniprot.org/uniprot/P00915) | Carbonic anhydrase 1 |
|  | [Q9UBD9](http://www.uniprot.org/uniprot/Q9UBD9) | Cardiotrophin-like cytokine factor 1 |
|  | [P07858](http://www.uniprot.org/uniprot/P07858) | Cathepsin B heavy chain |
|  | [Q8N6Q3](http://www.uniprot.org/uniprot/Q8N6Q3) | CD177 antigen |
|  | [P08962](http://www.uniprot.org/uniprot/P08962) | CD63 antigen |
|  | [P21926](http://www.uniprot.org/uniprot/P21926) | CD9 antigen |
|  | [P48960](http://www.uniprot.org/uniprot/P48960) | CD97 antigen subunit beta |
|  | [P36222](http://www.uniprot.org/uniprot/P36222) | Chitinase-3-like protein 1 |
|  | [Q13231](http://www.uniprot.org/uniprot/Q13231) | Chitotriosidase-1 |
|  | [Q8IWA5](http://www.uniprot.org/uniprot/Q8IWA5) | Choline transporter-like protein 2 |
|  | [P45973](http://www.uniprot.org/uniprot/P45973) | Chromobox protein homolog 5 |
|  | [P09496](http://www.uniprot.org/uniprot/P09496) | Clathrin light chain A |
|  | [P00748](http://www.uniprot.org/uniprot/P00748) | Coagulation factor XIIa light chain |
|  | [P23528](http://www.uniprot.org/uniprot/P23528) | Cofilin-1 |
|  | [Q9NZP8](http://www.uniprot.org/uniprot/Q9NZP8) | Complement C1r subcomponent-like protein |
|  | [P13671](http://www.uniprot.org/uniprot/P13671) | Complement component C6 |
|  | [P07357](http://www.uniprot.org/uniprot/P07357) | Complement component C8 alpha chain |
|  | [P07358](http://www.uniprot.org/uniprot/P07358) | Complement component C8 beta chain |
|  | [P07360](http://www.uniprot.org/uniprot/P07360) | Complement component C8 gamma chain |
|  | [P36980](http://www.uniprot.org/uniprot/P36980) | Complement factor H-related protein 2 |
|  | [Q13409](http://www.uniprot.org/uniprot/Q13409) | Cytoplasmic dynein 1 intermediate chain 2 |
|  | [P30046](http://www.uniprot.org/uniprot/P30046) | D-dopachrome decarboxylase |
|  | [P13716](http://www.uniprot.org/uniprot/P13716) | Delta-aminolevulinic acid dehydratase |
|  | [Q08554](http://www.uniprot.org/uniprot/Q08554) | Desmocollin-1 |
|  | [Q13822](http://www.uniprot.org/uniprot/Q13822) | Ectonucleotide pyrophosphatase/phosphodiesterase family member 2 |
|  | [Q9NZ08](http://www.uniprot.org/uniprot/Q9NZ08) | Endoplasmic reticulum aminopeptidase 1 |
|  | [Q9BS26](http://www.uniprot.org/uniprot/Q9BS26) | Endoplasmic reticulum resident protein 44 |
|  | [P27105](http://www.uniprot.org/uniprot/P27105) | Erythrocyte band 7 integral membrane protein |
|  | [P09972](http://www.uniprot.org/uniprot/P09972) | Fructose-bisphosphate aldolase C |
|  | [P17931](http://www.uniprot.org/uniprot/P17931) | Galectin-3 |
|  | [Q92820](http://www.uniprot.org/uniprot/Q92820) | Gamma-glutamyl hydrolase |
|  | [P17900](http://www.uniprot.org/uniprot/P17900) | Ganglioside GM2 activator isoform short |
|  | [P36269](http://www.uniprot.org/uniprot/P36269) | Glutathione hydrolase 5 light chain |
|  | [P20933](http://www.uniprot.org/uniprot/P20933) | Glycosylasparaginase beta chain |
|  | [Q92598](http://www.uniprot.org/uniprot/Q92598) | Heat shock protein 105 kDa |
|  | [O60506](http://www.uniprot.org/uniprot/O60506) | Heterogeneous nuclear ribonucleoprotein Q |
|  | [P22626](http://www.uniprot.org/uniprot/P22626) | Heterogeneous nuclear ribonucleoproteins A2/B1 |
|  | [Q71DI3](http://www.uniprot.org/uniprot/Q71DI3) | Histone H3.2 |
|  | [P01891](http://www.uniprot.org/uniprot/P01891) | HLA class I histocompatibility antigen, A-68 alpha chain |
|  | [Q31612](http://www.uniprot.org/uniprot/Q31612) | HLA class I histocompatibility antigen, B-73 alpha chain |
|  | [P04233](http://www.uniprot.org/uniprot/P04233) | HLA class II histocompatibility antigen gamma chain |
|  | [P01909](http://www.uniprot.org/uniprot/P01909) | HLA class II histocompatibility antigen, DQ alpha 1 chain |
|  | [P05538](http://www.uniprot.org/uniprot/P05538) | HLA class II histocompatibility antigen, DQ beta 2 chain |
|  | [Q30154](http://www.uniprot.org/uniprot/Q30154) | HLA class II histocompatibility antigen, DR beta 5 chain |
|  | [P01911](http://www.uniprot.org/uniprot/P01911) | HLA class II histocompatibility antigen, DRB1-15 beta chain |
|  | [P50502](http://www.uniprot.org/uniprot/P50502) | Hsc70-interacting protein |
|  | [Q9Y6R7](http://www.uniprot.org/uniprot/Q9Y6R7) | IgGFc-binding protein |
|  | [P01742](http://www.uniprot.org/uniprot/P01742) | Immunoglobulin heavy variable 1-69 |
|  | [P01825](http://www.uniprot.org/uniprot/P01825) | Immunoglobulin heavy variable 4-59 |
|  | [P01591](http://www.uniprot.org/uniprot/P01591) | Immunoglobulin J chain |
|  | [P01703](http://www.uniprot.org/uniprot/P01703) | Immunoglobulin lambda variable 1-40 |
|  | [A0A075B6K5](http://www.uniprot.org/uniprot/A0A075B6K5) | Immunoglobulin lambda variable 3-9 |
|  | [P32942](http://www.uniprot.org/uniprot/P32942) | Intercellular adhesion molecule 3 |
|  | [P18510](http://www.uniprot.org/uniprot/P18510) | Interleukin-1 receptor antagonist protein |
|  | [P53990](http://www.uniprot.org/uniprot/P53990) | IST1 homolog |
|  | [Q9Y624](http://www.uniprot.org/uniprot/Q9Y624) | Junctional adhesion molecule A |
|  | [P20700](http://www.uniprot.org/uniprot/P20700) | Lamin-B1 |
|  | [P12318](http://www.uniprot.org/uniprot/P12318) | Low affinity immunoglobulin gamma Fc region receptor II-a |
|  | [O00754](http://www.uniprot.org/uniprot/O00754) | Lysosomal alpha-mannosidase E peptide |
|  | [P42785](http://www.uniprot.org/uniprot/P42785) | Lysosomal Pro-X carboxypeptidase |
|  | [Q9UQV4](http://www.uniprot.org/uniprot/Q9UQV4) | Lysosome-associated membrane glycoprotein 3 |
|  | [P22897](http://www.uniprot.org/uniprot/P22897) | Macrophage mannose receptor 1 |
|  | [P14174](http://www.uniprot.org/uniprot/P14174) | Macrophage migration inhibitory factor |
|  | [P51674](http://www.uniprot.org/uniprot/P51674) | Neuronal membrane glycoprotein M6-a |
|  | [P05204](http://www.uniprot.org/uniprot/P05204) | Non-histone chromosomal protein HMG-17 |
|  | [Q9H1E3](http://www.uniprot.org/uniprot/Q9H1E3) | Nuclear ubiquitous casein and cyclin-dependent kinase substrate 1 |
|  | [O14638](http://www.uniprot.org/uniprot/O14638) | Nucleotide pyrophosphatase |
|  | [P49327](http://www.uniprot.org/uniprot/P49327) | Oleoyl-[acyl-carrier-protein] hydrolase |
|  | [P23284](http://www.uniprot.org/uniprot/P23284) | Peptidyl-prolyl cis-trans isomerase B |
|  | [P36871](http://www.uniprot.org/uniprot/P36871) | Phosphoglucomutase-1 |
|  | [Q96G03](http://www.uniprot.org/uniprot/Q96G03) | Phosphoglucomutase-2 |
|  | [P15291](http://www.uniprot.org/uniprot/P15291) | Processed beta-1,4-galactosyltransferase 1 |
|  | [Q9UL46](http://www.uniprot.org/uniprot/Q9UL46) | Proteasome activator complex subunit 2 |
|  | [P25787](http://www.uniprot.org/uniprot/P25787) | Proteasome subunit alpha type-2 |
|  | [P40306](http://www.uniprot.org/uniprot/P40306) | Proteasome subunit beta type-10 |
|  | [P49721](http://www.uniprot.org/uniprot/P49721) | Proteasome subunit beta type-2 |
|  | [P49720](http://www.uniprot.org/uniprot/P49720) | Proteasome subunit beta type-3 |
|  | [P28070](http://www.uniprot.org/uniprot/P28070) | Proteasome subunit beta type-4 |
|  | [P28074](http://www.uniprot.org/uniprot/P28074) | Proteasome subunit beta type-5 |
|  | [P28062](http://www.uniprot.org/uniprot/P28062) | Proteasome subunit beta type-8 |
|  | [P07237](http://www.uniprot.org/uniprot/P07237) | Protein disulfide-isomerase |
|  | [Q9NUQ9](http://www.uniprot.org/uniprot/Q9NUQ9) | Protein FAM49B |
|  | [Q92954](http://www.uniprot.org/uniprot/Q92954) | Proteoglycan 4 C-terminal part |
|  | [P00491](http://www.uniprot.org/uniprot/P00491) | Purine nucleoside phosphorylase |
|  | [P50395](http://www.uniprot.org/uniprot/P50395) | Rab GDP dissociation inhibitor beta |
|  | [Q13283](http://www.uniprot.org/uniprot/Q13283) | Ras GTPase-activating protein-binding protein 1 |
|  | [P61026](http://www.uniprot.org/uniprot/P61026) | Ras-related protein Rab-10 |
|  | [P61106](http://www.uniprot.org/uniprot/P61106) | Ras-related protein Rab-14 |
|  | [P62820](http://www.uniprot.org/uniprot/P62820) | Ras-related protein Rab-1A |
|  | [P61020](http://www.uniprot.org/uniprot/P61020) | Ras-related protein Rab-5B |
|  | [P11233](http://www.uniprot.org/uniprot/P11233) | Ras-related protein Ral-A |
|  | [Q12913](http://www.uniprot.org/uniprot/Q12913) | Receptor-type tyrosine-protein phosphatase eta |
|  | [Q99969](http://www.uniprot.org/uniprot/Q99969) | Retinoic acid receptor responder protein 2 |
|  | [O75326](http://www.uniprot.org/uniprot/O75326) | Semaphorin-7A |
|  | [Q86VE9](http://www.uniprot.org/uniprot/Q86VE9) | Serine incorporator 5 |
|  | [Q9BRF8](http://www.uniprot.org/uniprot/Q9BRF8) | Serine/threonine-protein phosphatase CPPED1 |
|  | [Q96P63](http://www.uniprot.org/uniprot/Q96P63) | Serpin B12 |
|  | [P50453](http://www.uniprot.org/uniprot/P50453) | Serpin B9 |
|  | [P02743](http://www.uniprot.org/uniprot/P02743) | Serum amyloid P-component(1-203) |
|  | [Q9BZZ2](http://www.uniprot.org/uniprot/Q9BZZ2) | Sialoadhesin |
|  | [Q8WVQ1](http://www.uniprot.org/uniprot/Q8WVQ1) | Soluble calcium-activated nucleotidase 1 |
|  | [Q86VB7](http://www.uniprot.org/uniprot/Q86VB7) | Soluble CD163 |
|  | [P04004](http://www.uniprot.org/uniprot/P04004) | Somatomedin-B |
|  | [O00391](http://www.uniprot.org/uniprot/O00391) | Sulfhydryl oxidase 1 |
|  | [Q9UGT4](http://www.uniprot.org/uniprot/Q9UGT4) | Sushi domain-containing protein 2 |
|  | [P50990](http://www.uniprot.org/uniprot/P50990) | T-complex protein 1 subunit theta |
|  | [P35443](http://www.uniprot.org/uniprot/P35443) | Thrombospondin-4 |
|  | [P37837](http://www.uniprot.org/uniprot/P37837) | Transaldolase |
|  | [P20061](http://www.uniprot.org/uniprot/P20061) | Transcobalamin-1 |
|  | [P13693](http://www.uniprot.org/uniprot/P13693) | Translationally-controlled tumor protein |
|  | [P02652](http://www.uniprot.org/uniprot/P02652) | Truncated apolipoprotein A-II |
|  | [P02760](http://www.uniprot.org/uniprot/P02760) | Trypstatin |
|  | [P55327](http://www.uniprot.org/uniprot/P55327) | Tumor protein D52 |
|  | [P78324](http://www.uniprot.org/uniprot/P78324) | Tyrosine-protein phosphatase non-receptor type substrate 1 |
|  | [P61086](http://www.uniprot.org/uniprot/P61086) | Ubiquitin-conjugating enzyme E2 K |
|  | [Q13404](http://www.uniprot.org/uniprot/Q13404) | Ubiquitin-conjugating enzyme E2 variant 1 |
|  | [P00749](http://www.uniprot.org/uniprot/P00749) | Urokinase-type plasminogen activator chain B |
|  | [P19320](http://www.uniprot.org/uniprot/P19320) | Vascular cell adhesion protein 1 |
|  | [P08670](http://www.uniprot.org/uniprot/P08670) | Vimentin |
|  | [P02774](http://www.uniprot.org/uniprot/P02774) | Vitamin D-binding protein |
| **Neutrophil activation involved in immune response (GO:0002283) and Neutrophil degranulation (GO:0043312) 14.8%** | [Q01518](http://www.uniprot.org/uniprot/Q01518) | Adenylyl cyclase-associated protein 1 |
|  | [P43353](http://www.uniprot.org/uniprot/P43353) | Aldehyde dehydrogenase family 3 member B1 |
|  | [P19652](http://www.uniprot.org/uniprot/P19652) | Alpha-1-acid glycoprotein 2 |
|  | [P01011](http://www.uniprot.org/uniprot/P01011) | Alpha-1-antichymotrypsin His-Pro-less |
|  | [P04217](http://www.uniprot.org/uniprot/P04217) | Alpha-1B-glycoprotein |
|  | [P02765](http://www.uniprot.org/uniprot/P02765) | Alpha-2-HS-glycoprotein chain B |
|  | [P07355](http://www.uniprot.org/uniprot/P07355) | Annexin A2 |
|  | [P12429](http://www.uniprot.org/uniprot/P12429) | Annexin A3 |
|  | [P61769](http://www.uniprot.org/uniprot/P61769) | Beta-2-microglobulin form pI 5.3 |
|  | [P80511](http://www.uniprot.org/uniprot/P80511) | Calcitermin |
|  | [P04040](http://www.uniprot.org/uniprot/P04040) | Catalase |
|  | [P09668](http://www.uniprot.org/uniprot/P09668) | Cathepsin H light chain |
|  | [P11717](http://www.uniprot.org/uniprot/P11717) | Cation-independent mannose-6-phosphate receptor |
|  | [P16070](http://www.uniprot.org/uniprot/P16070) | CD44 antigen |
|  | [Q14019](http://www.uniprot.org/uniprot/Q14019) | Coactosin-like protein |
|  | [P04080](http://www.uniprot.org/uniprot/P04080) | Cystatin-B |
|  | [P01034](http://www.uniprot.org/uniprot/P01034) | Cystatin-C |
|  | [P15924](http://www.uniprot.org/uniprot/P15924) | Desmoplakin |
|  | [P68104](http://www.uniprot.org/uniprot/P68104) | Elongation factor 1-alpha 1 |
|  | [P13639](http://www.uniprot.org/uniprot/P13639) | Elongation factor 2 |
|  | [Q01469](http://www.uniprot.org/uniprot/Q01469) | Fatty acid-binding protein 5 |
|  | [P02794](http://www.uniprot.org/uniprot/P02794) | Ferritin heavy chain, N-terminally processed |
|  | [P02792](http://www.uniprot.org/uniprot/P02792) | Ferritin light chain |
|  | [P04075](http://www.uniprot.org/uniprot/P04075) | Fructose-bisphosphate aldolase A |
|  | [P06396](http://www.uniprot.org/uniprot/P06396) | Gelsolin |
|  | [O43451](http://www.uniprot.org/uniprot/O43451) | Glucoamylase |
|  | [P06744](http://www.uniprot.org/uniprot/P06744) | Glucose-6-phosphate isomerase |
|  | [P11142](http://www.uniprot.org/uniprot/P11142) | Heat shock cognate 71 kDa protein |
|  | [P07900](http://www.uniprot.org/uniprot/P07900) | Heat shock protein HSP 90-alpha |
|  | [O75874](http://www.uniprot.org/uniprot/O75874) | Isocitrate dehydrogenase [NADP] cytoplasmic |
|  | [P02788](http://www.uniprot.org/uniprot/P02788) | Lactoferroxin-C |
|  | [P02750](http://www.uniprot.org/uniprot/P02750) | Leucine-rich alpha-2-glycoprotein |
|  | [P30740](http://www.uniprot.org/uniprot/P30740) | Leukocyte elastase inhibitor |
|  | [P09960](http://www.uniprot.org/uniprot/P09960) | Leukotriene A-4 hydrolase |
|  | [P08571](http://www.uniprot.org/uniprot/P08571) | Monocyte differentiation antigen CD14, membrane-bound form |
|  | [P80188](http://www.uniprot.org/uniprot/P80188) | Neutrophil gelatinase-associated lipocalin |
|  | [P61916](http://www.uniprot.org/uniprot/P61916) | NPC intracellular cholesterol transporter 2 |
|  | [O95497](http://www.uniprot.org/uniprot/O95497) | Pantetheinase |
|  | [P62937](http://www.uniprot.org/uniprot/P62937) | Peptidyl-prolyl cis-trans isomerase A, N-terminally processed |
|  | [P30041](http://www.uniprot.org/uniprot/P30041) | Peroxiredoxin-6 |
|  | [P18669](http://www.uniprot.org/uniprot/P18669) | Phosphoglycerate mutase 1 |
|  | [P31949](http://www.uniprot.org/uniprot/P31949) | Protein S100-A11, N-terminally processed |
|  | [P14618](http://www.uniprot.org/uniprot/P14618) | Pyruvate kinase PKM |
|  | [P07602](http://www.uniprot.org/uniprot/P07602) | Saposin-D |
|  | [P01833](http://www.uniprot.org/uniprot/P01833) | Secretory component |
|  | [P01009](http://www.uniprot.org/uniprot/P01009) | Short peptide from AAT |
|  | [Q13813](http://www.uniprot.org/uniprot/Q13813) | Spectrin alpha chain, non-erythrocytic 1 |
|  | [P02766](http://www.uniprot.org/uniprot/P02766) | Transthyretin |
|  | [P18206](http://www.uniprot.org/uniprot/P18206) | Vinculin |
| **Myeloid leukocyte activation (GO:0002274) 7%** | [P02765](http://www.uniprot.org/uniprot/P02765) | Alpha-2-HS-glycoprotein chain B |
|  | [P37840](http://www.uniprot.org/uniprot/P37840) | Alpha-synuclein |
|  | [P07686](http://www.uniprot.org/uniprot/P07686) | Beta-hexosaminidase subunit beta chain A |
|  | [P07339](http://www.uniprot.org/uniprot/P07339) | Cathepsin D heavy chain |
|  | [P25774](http://www.uniprot.org/uniprot/P25774) | Cathepsin S |
|  | [Q02413](http://www.uniprot.org/uniprot/Q02413) | Desmoglein-1 |
|  | [P53634](http://www.uniprot.org/uniprot/P53634) | Dipeptidyl peptidase 1 light chain |
|  | [P08238](http://www.uniprot.org/uniprot/P08238) | Heat shock protein HSP 90-beta |
|  | [P09429](http://www.uniprot.org/uniprot/P09429) | High mobility group protein B1 |
|  | [P14923](http://www.uniprot.org/uniprot/P14923) | Junction plakoglobin |
|  | [P02750](http://www.uniprot.org/uniprot/P02750) | Leucine-rich alpha-2-glycoprotein |
|  | [P18428](http://www.uniprot.org/uniprot/P18428) | Lipopolysaccharide-binding protein |
|  | [P11279](http://www.uniprot.org/uniprot/P11279) | Lysosome-associated membrane glycoprotein 1 |
|  | [P61626](http://www.uniprot.org/uniprot/P61626) | Lysozyme C |
|  | [P28066](http://www.uniprot.org/uniprot/P28066) | Proteasome subunit alpha type-5 |
|  | [Q99436](http://www.uniprot.org/uniprot/Q99436) | Proteasome subunit beta type-7 |
|  | [Q99584](http://www.uniprot.org/uniprot/Q99584) | Protein S100-A13 |
|  | [P46940](http://www.uniprot.org/uniprot/P46940) | Ras GTPase-activating-like protein IQGAP1 |
|  | [P48595](http://www.uniprot.org/uniprot/P48595) | Serpin B10 |
|  | [P35237](http://www.uniprot.org/uniprot/P35237) | Serpin B6 |
|  | [P68871](http://www.uniprot.org/uniprot/P68871) | Spinorphin |
|  | [P37173](http://www.uniprot.org/uniprot/P37173) | TGF-beta receptor type-2 |
|  | [P55072](http://www.uniprot.org/uniprot/P55072) | Transitional endoplasmic reticulum ATPase |
| **Complement activation (GO:0006956) 6.3%** | [P04003](http://www.uniprot.org/uniprot/P04003) | C4b-binding protein alpha chain |
|  | [P10909](http://www.uniprot.org/uniprot/P10909) | Clusterin alpha chain |
|  | [P02746](http://www.uniprot.org/uniprot/P02746) | Complement C1q subcomponent subunit B |
|  | [P02747](http://www.uniprot.org/uniprot/P02747) | Complement C1q subcomponent subunit C |
|  | [P00736](http://www.uniprot.org/uniprot/P00736) | Complement C1r subcomponent light chain |
|  | [P09871](http://www.uniprot.org/uniprot/P09871) | Complement C1s subcomponent light chain |
|  | [P06681](http://www.uniprot.org/uniprot/P06681) | Complement C2a fragment |
|  | [P01024](http://www.uniprot.org/uniprot/P01024) | Complement C3c alpha' chain fragment 2 |
|  | [P0C0L4](http://www.uniprot.org/uniprot/P0C0L4) | Complement C4 gamma chain |
|  | [P01031](http://www.uniprot.org/uniprot/P01031) | Complement C5 alpha' chain |
|  | [P10643](http://www.uniprot.org/uniprot/P10643) | Complement component C7 |
|  | [P02748](http://www.uniprot.org/uniprot/P02748) | Complement component C9b |
|  | [P00751](http://www.uniprot.org/uniprot/P00751) | Complement factor B Bb fragment |
|  | [P00746](http://www.uniprot.org/uniprot/P00746) | Complement factor D |
|  | [P08603](http://www.uniprot.org/uniprot/P08603) | Complement factor H |
|  | [P05156](http://www.uniprot.org/uniprot/P05156) | Complement factor I light chain |
|  | [O75636](http://www.uniprot.org/uniprot/O75636) | Ficolin-3 |
|  | [P01854](http://www.uniprot.org/uniprot/P01854) | Immunoglobulin heavy constant epsilon |
|  | [P01871](http://www.uniprot.org/uniprot/P01871) | Immunoglobulin heavy constant mu |
|  | [P05155](http://www.uniprot.org/uniprot/P05155) | Plasma protease C1 inhibitor |
|  | [P27918](http://www.uniprot.org/uniprot/P27918) | Properdin |
| **Activation of immune response (GO:0002253) 4.5%** | [P10809](http://www.uniprot.org/uniprot/P10809) | 60 kDa heat shock protein, mitochondrial |
|  | [P04114](http://www.uniprot.org/uniprot/P04114) | Apolipoprotein B-48 |
|  | [P07711](http://www.uniprot.org/uniprot/P07711) | Cathepsin L1 light chain |
|  | [P14625](http://www.uniprot.org/uniprot/P14625) | Endoplasmin |
|  | [Q9HC84](http://www.uniprot.org/uniprot/Q9HC84) | Mucin-5B |
|  | [Q06323](http://www.uniprot.org/uniprot/Q06323) | Proteasome activator complex subunit 1 |
|  | [P25786](http://www.uniprot.org/uniprot/P25786) | Proteasome subunit alpha type-1 |
|  | [P25788](http://www.uniprot.org/uniprot/P25788) | Proteasome subunit alpha type-3 |
|  | [P25789](http://www.uniprot.org/uniprot/P25789) | Proteasome subunit alpha type-4 |
|  | [P60900](http://www.uniprot.org/uniprot/P60900) | Proteasome subunit alpha type-6 |
|  | [O14818](http://www.uniprot.org/uniprot/O14818) | Proteasome subunit alpha type-7 |
|  | [P20618](http://www.uniprot.org/uniprot/P20618) | Proteasome subunit beta type-1 |
|  | [P28072](http://www.uniprot.org/uniprot/P28072) | Proteasome subunit beta type-6 |
|  | [Q8IWL2](http://www.uniprot.org/uniprot/Q8IWL2) | Pulmonary surfactant-associated protein A1 |
|  | [P61088](http://www.uniprot.org/uniprot/P61088) | Ubiquitin-conjugating enzyme E2 N |
| **Innate immune response (GO:0045087) 3.6%** | [Q15109](http://www.uniprot.org/uniprot/Q15109) | Advanced glycosylation end product-specific receptor |
|  | [P49913](http://www.uniprot.org/uniprot/P49913) | Antibacterial peptide LL-37 |
|  | [P06727](http://www.uniprot.org/uniprot/P06727) | Apolipoprotein A-IV |
|  | [P08174](http://www.uniprot.org/uniprot/P08174) | Complement decay-accelerating factor |
|  | [Q9UGM3](http://www.uniprot.org/uniprot/Q9UGM3) | Deleted in malignant brain tumors 1 protein |
|  | [P26583](http://www.uniprot.org/uniprot/P26583) | High mobility group protein B2 |
|  | [P07333](http://www.uniprot.org/uniprot/P07333) | Macrophage colony-stimulating factor 1 receptor |
|  | [Q9UEW3](http://www.uniprot.org/uniprot/Q9UEW3) | Macrophage receptor MARCO |
|  | [P06702](http://www.uniprot.org/uniprot/P06702) | Protein S100-A9 |
|  | [O00584](http://www.uniprot.org/uniprot/O00584) | Ribonuclease T2 |
|  | [P68371](http://www.uniprot.org/uniprot/P68371) | Tubulin beta-4B chain |
|  | [Q14508](http://www.uniprot.org/uniprot/Q14508) | WAP four-disulfide core domain protein 2 |
| **Platelet degranulation (GO:0002576) 3.6%** | [Q14624](http://www.uniprot.org/uniprot/Q14624) | 35 kDa inter-alpha-trypsin inhibitor heavy chain H4 |
|  | [O43707](http://www.uniprot.org/uniprot/O43707) | Alpha-actinin-4 |
|  | [Q6YHK3](http://www.uniprot.org/uniprot/Q6YHK3) | CD109 antigen |
|  | [Q16610](http://www.uniprot.org/uniprot/Q16610) | Extracellular matrix protein 1 |
|  | [Q08380](http://www.uniprot.org/uniprot/Q08380) | Galectin-3-binding protein |
|  | [P02787](http://www.uniprot.org/uniprot/P02787) | Serotransferrin |
|  | [P02768](http://www.uniprot.org/uniprot/P02768) | Serum albumin |
|  | [P09486](http://www.uniprot.org/uniprot/P09486) | SPARC |
|  | [P05452](http://www.uniprot.org/uniprot/P05452) | Tetranectin |
|  | [P37802](http://www.uniprot.org/uniprot/P37802) | Transgelin-2 |
|  | [P02647](http://www.uniprot.org/uniprot/P02647) | Truncated apolipoprotein A-I |
|  | [O75083](http://www.uniprot.org/uniprot/O75083) | WD repeat-containing protein 1 |
| **Negative regulation of blood coagulation (GO:0030195) 3.3%** | [P08697](http://www.uniprot.org/uniprot/P08697) | Alpha-2-antiplasmin |
|  | [P02649](http://www.uniprot.org/uniprot/P02649) | Apolipoprotein E |
|  | [P02749](http://www.uniprot.org/uniprot/P02749) | Beta-2-glycoprotein 1 |
|  | [P02671](http://www.uniprot.org/uniprot/P02671) | Fibrinogen alpha chain |
|  | [P02675](http://www.uniprot.org/uniprot/P02675) | Fibrinogen beta chain |
|  | [P02679](http://www.uniprot.org/uniprot/P02679) | Fibrinogen gamma chain |
|  | [P04196](http://www.uniprot.org/uniprot/P04196) | Histidine-rich glycoprotein |
|  | [P01042](http://www.uniprot.org/uniprot/P01042) | Low molecular weight growth-promoting factor |
|  | [P00747](http://www.uniprot.org/uniprot/P00747) | Plasmin light chain B |
|  | [P00734](http://www.uniprot.org/uniprot/P00734) | Thrombin heavy chain |
|  | [P08758](http://www.uniprot.org/uniprot/P08758) | Annexin A5 |
| **Inflammatory response (GO: 0006954) 1.8%** | [O75882](http://www.uniprot.org/uniprot/O75882) | Attractin |
|  | [P00739](http://www.uniprot.org/uniprot/P00739) | Haptoglobin-related protein |
|  | [P05362](http://www.uniprot.org/uniprot/P05362) | Intercellular adhesion molecule 1 |
|  | [Q9NPH3](http://www.uniprot.org/uniprot/Q9NPH3) | Interleukin-1 receptor accessory protein |
|  | [Q16853](http://www.uniprot.org/uniprot/Q16853) | Membrane primary amine oxidase |
|  | [P30044](http://www.uniprot.org/uniprot/P30044) | Peroxiredoxin-5, mitochondrial |
| **Regulation of immune system process (GO:0002682) 1.5%** | [P15311](http://www.uniprot.org/uniprot/P15311) | Ezrin |
|  | [P02790](http://www.uniprot.org/uniprot/P02790) | Hemopexin |
|  | [P26038](http://www.uniprot.org/uniprot/P26038) | Moesin |
|  | [P0CG48](http://www.uniprot.org/uniprot/P0CG48) | Ubiquitin |
|  | [P25311](http://www.uniprot.org/uniprot/P25311) | Zinc-alpha-2-glycoprotein |
| **Regulation of inflammatory response (GO:0050727) 1.5%** | [P01023](http://www.uniprot.org/uniprot/P01023) | Alpha-2-macroglobulin |
|  | [P01019](http://www.uniprot.org/uniprot/P01019) | Angiotensin 1-4 |
|  | [P09211](http://www.uniprot.org/uniprot/P09211) | Glutathione S-transferase P |
|  | [P62328](http://www.uniprot.org/uniprot/P62328) | Hematopoietic system regulatory peptide |
|  | [P36955](http://www.uniprot.org/uniprot/P36955) | Pigment epithelium-derived factor |
| **Platelet aggregation (GO:0070527) 1.5%** | [P60709](http://www.uniprot.org/uniprot/P60709) | Actin, cytoplasmic 1, N-terminally processed |
|  | [P21333](http://www.uniprot.org/uniprot/P21333) | Filamin-A |
|  | [P04792](http://www.uniprot.org/uniprot/P04792) | Heat shock protein beta-1 |
|  | [P35579](http://www.uniprot.org/uniprot/P35579) | Myosin-9 |
|  | [P02751](http://www.uniprot.org/uniprot/P02751) | Ugl-Y3 |
| **Phagocytosis, engulfment (GO:0006911) 1.2%** | [P01877](http://www.uniprot.org/uniprot/P01877) | Immunoglobulin heavy constant alpha 2 |
|  | [P01857](http://www.uniprot.org/uniprot/P01857) | Immunoglobulin heavy constant gamma 1 |
|  | [P01834](http://www.uniprot.org/uniprot/P01834) | Immunoglobulin kappa constant |
|  | [B9A064](http://www.uniprot.org/uniprot/B9A064) | Immunoglobulin lambda-like polypeptide 5 |
| **Regulation of defense response (GO:0031347) 1.2%** | [Q15848](http://www.uniprot.org/uniprot/Q15848) | Adiponectin |
|  | [P33151](http://www.uniprot.org/uniprot/P33151) | Cadherin-5 |
|  | [Q9BY67](http://www.uniprot.org/uniprot/Q9BY67) | Cell adhesion molecule 1 |
|  | [P21980](http://www.uniprot.org/uniprot/P21980) | Protein-glutamine gamma-glutamyltransferase 2 |
| **Regulation of lymphocyte activation (GO:0051249) 1.2%** | [P04083](http://www.uniprot.org/uniprot/P04083) | Annexin A1 |
|  | [P35247](http://www.uniprot.org/uniprot/P35247) | Pulmonary surfactant-associated protein D |
|  | [P00441](http://www.uniprot.org/uniprot/P00441) | Superoxide dismutase [Cu-Zn] |
|  | [P11684](http://www.uniprot.org/uniprot/P11684) | Uteroglobin |
| **Antimicrobial humoral response (GO:0019730) 0.9%** | [Q8TDL5](http://www.uniprot.org/uniprot/Q8TDL5) | BPI fold-containing family B member 1 |
|  | [P04406](http://www.uniprot.org/uniprot/P04406) | Glyceraldehyde-3-phosphate dehydrogenase |
|  | [P62807](http://www.uniprot.org/uniprot/P62807) | Histone H2B type 1-C/E/F/G/I |
| **Regulation of acute inflammatory response (GO: 0002673) 0.6%** | [P40189](http://www.uniprot.org/uniprot/P40189) | Interleukin-6 receptor subunit beta |
|  | [Q99497](http://www.uniprot.org/uniprot/Q99497) | Protein/nucleic acid deglycase DJ-1 |
| **Regulation of complement activation (GO:0030449) 0.6%** | [O43866](http://www.uniprot.org/uniprot/O43866) | CD5 antigen-like |
|  | [P07225](http://www.uniprot.org/uniprot/P07225) | Vitamin K-dependent protein S |
| **Regulation of B cell activation (GO:0050864) 0.3%** | [B9A064](http://www.uniprot.org/uniprot/B9A064) | Immunoglobulin lambda-like polypeptide 5 |
| **Regulation of coagulation (GO:0050818) 0.3%** | [P01008](http://www.uniprot.org/uniprot/P01008) | Antithrombin-III |
